# Expansion of Multipotent Stem Cells from the Adult Human Brain

**DOI:** 10.1371/journal.pone.0071334

**Published:** 2013-08-14

**Authors:** Wayne Murrell, Emily Palmero, John Bianco, Biljana Stangeland, Mrinal Joel, Linda Paulson, Bernd Thiede, Zanina Grieg, Ingunn Ramsnes, Håvard K. Skjellegrind, Ståle Nygård, Petter Brandal, Cecilie Sandberg, Einar Vik-Mo, Sheryl Palmero, Iver A. Langmoen

**Affiliations:** 1 Vilhelm Magnus Laboratory for Neurosurgical Research, Institute for Surgical Research, Oslo University Hospital, Oslo, Norway; 2 The Biotechnology Centre of Oslo, University of Oslo, Oslo, Norway; 3 Research Group for Biomedical Informatics, University of Oslo, Oslo, Norway; 4 Center for Cancer Biomedicine, The Norwegian Radium Hospital, Oslo, Norway; 5 Department of Neurosurgery, Oslo University Hospital, Oslo, Norway; 6 Norwegian Center for Stem Cell Research, Oslo University Hospital, Oslo, Norway; University of Nebraska Medical Center, United States of America

## Abstract

The discovery of stem cells in the adult human brain has revealed new possible scenarios for treatment of the sick or injured brain. Both clinical use of and preclinical research on human adult neural stem cells have, however, been seriously hampered by the fact that it has been impossible to passage these cells more than a very few times and with little expansion of cell numbers. Having explored a number of alternative culturing conditions we here present an efficient method for the establishment and propagation of human brain stem cells from whatever brain tissue samples we have tried. We describe virtually unlimited expansion of an authentic stem cell phenotype. Pluripotency proteins Sox2 and Oct4 are expressed without artificial induction. For the first time multipotency of adult human brain-derived stem cells is demonstrated beyond tissue boundaries. We characterize these cells in detail *in vitro* including microarray and proteomic approaches. Whilst clarification of these cells’ behavior is ongoing, results so far portend well for the future repair of tissues by transplantation of an adult patient’s own-derived stem cells.

## Introduction

A scenario that has captured the imagination is the potential advent of tissue repair using cell manipulation and transplantation. In reality surgical intervention has already made pioneering inroads using cell transplant. Bone marrow reconstitution commenced in 1956 with ED Thomas’ pioneering work [1], [2]. Today cellular colonization of extracellular matrix scaffolds has been employed to replace organ and complex tissue structures in trachea, bladder, muscle and bone [3]. Emulating the 3-D structure of extracellular matrix scaffolds with artificial nanofibre matrices takes tissue repair to a new frontier [4]. These interventions utilize autologous cell grafts. Understanding the biology of resident stem and progenitor cells found in human organs is therefore a prerequisite platform of knowledge.

Our laboratory works on the human brain. We have access to a continuing supply of adult human brain samples from both diseased and ‘normal’ people. Already our lab has investigated harvest, culture and differentiation of human central nervous system (CNS)-derived cells [5], [6], [7], [8], [9], [10], [11]. For these experiments we have employed methods originally derived from well established rodent protocols for the ‘neurosphere assay’ [12], [13]. Some labs have published studies of human brain stem cell culture but these are limited and the varying methods have not become routine [14], [15], [16], [17], [18], [19], [20], [21]. There is little published data quantitating successful long term propagation of adult human brain progenitors. Our own former studies have been hampered by the difficulty of expanding human neural progenitors thereby limiting the scope of experiments and therefore as well the possible exploration of tissue repair potential.

The adult human brain contains stem cells that can differentiate into mature neurons that generate action potentials [5], [11] and communicate by synapses [6]. It is expected that these cells in the future may be used to treat neurodegenerative diseases (e.g. Parkinsońs disease) and brain injuries. Because it has, however, turned out that stem cells from the adult human brain are very hard to expand both preclinical research and putative clinical applications have been impeded by low cell numbers, and progress in this otherwise promising field has been slow.

We hypothesized that proliferative limitation could be overcome by exploration of variant or alternative culturing conditions. We here present a fail-safe method for successful culture and expansion of human brain progenitors. This method overturns the belief that neural stem cells need to be cultured as neurospheres and has allowed for the establishment and propagation of human brain progenitor cells from whatever brain tissue samples we have tried. We have achieved virtually unlimited expansion of the cells which raises questions pertinent to developing transplant protocols. What is the phenotype of these cells? Does the phenotype and potential vary according to cell source? Is that phenotype stable over the period of expansion? Are they stem or progenitor cells? Are they transformed and potentially able to form tumors? Can they be differentiated *in vitro* and *in vivo*? Could they be used to make dopaminergic neurons for instance?

We show these adult human brain-derived cells express pluripotency proteins Sox2 and Oct4 without artificial induction and for the first time offer data strongly supportive of their multipotency well beyond tissue boundaries when transplanted into chick embryos. This was shown for mouse brain-derived stem cells [22]but not human until now. We have investigated the above issues extensively and assessed phenotype of the various populations using immunochemistry, microarray, proteomics and western blot.

We do not claim that these brain-derived stem cells are pluripotent but they could be. That would require demonstration of the acquisition of every possible cell phenotype in the body. These cells are ‘neural stem cells’. We define a neural stem cell as a stem cell derived from any part of the nervous system and which primarily makes cells expressing neural markers (those of astrocytes, oligodendrocytes and neurons) in *in vitro* culture. In order to verify that the cultures analysed truly contain stem cells we sought to show that they satisfied the following criteria:

- A stem cell is self renewing.
- A stem cell can make many cell types.
- A stem cell can be cloned and thence renew and be able to make multiple cell types.
- A stem cell expresses protein markers known to be associated with ‘stemness’.
- A stem cell responds to the tissue milieu of signals it receives.

## Materials and Methods

Ventricular wall biopsies were obtained from temporal lobe specimens removed due to medically refractory epilepsy. Tissue was obtained from consenting patients, ranging in age from 23 to 62 years. Tissue harvesting was approved by the Norwegian National Committee for Medical Research Ethics. Tumor biopsy specimens were obtained from informed and consenting patients, and the tissue harvesting was approved by the Norwegian National Committee for Medical Research Ethics (07321b). All animal procedures were approved by the National Animal Research Authority (1094/2008).

The biopsies were transported from the operating theatre in Leibowitz-15 medium (L15, Invitrogen, Carlsbad, CA), stored at +4°C and initially cultured as described previously [5], [10]. Surgical specimens have been described as the following sources: hippocampus (HPC), subventricular zone (SVZ), white matter (WM), grey matter (GM), cortex, paraventricular zone and mixtures of these. For the most part data presented in this paper emanate from HPC, SVZ, WM and GM-derived cultures only.

### Suspension Cell Culture

Tissue samples were minced with a McIlwain Tissue Chopper (Mickle Laboratory Engineering, UK) and digested in 20 units/mL papain (Worthington, NJ) incubated at 37°C for five minutes. The digestions were stopped by the addition of 10 mL DPBS (Dulbecco’s Phosphate Buffered Saline, Lonza, BioWhittaker) with 1% vol/vol albumin (Octapharma AS). The pellets collected by centrifugation at 300 g for five min at room temperature (RT) were resuspended in DMEM/F12 and counted using a NucleoCounter (Chemometec, Denmark) (to exclude dead cells) and cultured as described previously [5], [10]. The cells were seeded at a density of 4000 cells/cm^2^ ie. 20,000 cells/mL in DMEM/F12 (Invitrogen) supplemented with 2% B27-without retinoic acid (RA, Invitrogen), 10 mM hepes (Lonza, BioWhittaker), 2.5 µg/mL heparin (LeoPharma AS) and 1% penicillin/streptomycin (Lonza, BioWhittaker) in non-treated flasks (Nunc, VWR) at 37°C in 5% CO_2_.

The cultures were supplemented with 10 ng/mL basic fibroblast growth factor (bFGF, R&D Inc, Minneapolis) and 20 ng/mL epidermal growth factor (EGF, R&D Inc) three times a week that resulted in formation of floating aggregates (neurospheres) and additional DMEM/F12 was added once a week [10]. For subsequent neurosphere formation (cell passage), the neurospheres were dissociated into single cells when they reached 12–15 cells in diameter, by incubating in papain followed by mechanical resuspension and cultured further in the presence of the aforementioned mitogens. This suspension culture method is the standard method used to propagate tumor-derived spheres in our laboratory [7], [8], [9], [23], [24] and is called ‘VML’ culture protocol.

### Additional Components Tested

These included N2 Supplement (1×); 10% and 5% FBS; LIF, 10 ng/mL; EGF, 50 and 25 ng/mL; bFGF, 50 and 25 ng/mL; TGFα, 20 ng/mL; Bovine pituitary extract, 30 µg/mL; Forskolin, 40 ng/mL; IBMX (3-Isobutyl-1-methylxanthine), 50 and 250 µM; laminin, 1 µg/mL; FGF8, 50 ng/mL; Shh (Sonic hedgehog), 500 ng/mL; poly-L-lysine, 1 µg/cm2; and Xanthosine, 400 and 200 µM.

### Making Neurospheres from Adherent Culture

An optimal method was found to be 1 µg/cm^2^ poly-L-lysine, 2% vol/vol B27 without RA, 20 ng/mL EGF, 10 ng/mL bFGF, 2.5 µg/mL heparin, 10 mM hepes, 30 µg/mL bovine pituitary extract (BPE) and 1% P/S. Under these conditions abundant neurospheres appeared in two days that eventually detached and floated off.

### Attempts to Differentiate Dopaminergic Neurons

Cells, from dissociated neurospheres or adherent cultures were resuspended in DMEM/ITS (ITS: 1 g/L insulin, 0.55 g/L transferrin, 0.67 mg/L sodium selenite) with or without GDNF (25 ng/mL; Chemicon, Temecula, CA, http://www.chemicon.com) and plated into plastic multiwell plates (Nunc). The wells were uncoated or coated with either collagen IV alone or with poly-L-ornithine plus laminin. Collagen IV (5 µg/cm^2^; Sigma-Aldrich) was applied in sterile H_2_O and allowed to dry overnight at room temperature. Poly-L-ornithine (28 µg/cm^2^, 0.1 mg/mL; Sigma-Aldrich) was incubated overnight in sterile H_2_O and then washed three times in HBSS prior to laminin coating (2.9 µg/cm^2^, 10 µg/mL; Gibco) in sterile H_2_O. As well different concentrations of FBS were included.

### Protocol for Immunohistochemical Labeling in Plastic Multiwell Plates

Cells were rinsed gently in PBS or HBSS and then fixed in 4% paraformaldehyde (PFA) in PBS pH 7.4, for 10 min at RT. Fixative was washed off with PBS three times. Cells were permeablised and blocked for non-specific binding with 10% serum of the animal the 2° antibody was raised in in PBS pH 7.4, 1% Triton X-100, 0.3% H_2_O_2_ (to quench endogenous peroxidases) for 1 h, at RT, on a shaker. After two washes they were incubated in 1° antibody, 2% serum in PBS, pH 7.4 for 1 hour (h) at RT on a shaker. They were then washed twice in PBS, 0.1% Tween 20, 1% serum, for 10 min, on a shaker. This was followed then by a wash omitting the Tween 20. Biotinylated secondary antibodies (Vector Labs) were then applied for 30 min, at RT in 1% serum in PBS on a shaker. Washing and detection using ABC reagent (avidin-biotin-complex) were performed according to the manufacturer’s instructions (Vector Labs). Plates were stored in 0.1% azide in PBS at 4°C.

### Antibodies used for Immunohistochemical Labeling

Rabbit anti-Gal C, 1/200 (Sigma); rabbit anti-GFAP, 1/200 (DakoCytomation, Denmark); rabbit anti-Integrin β1, 1/200 (Abcam); rabbit anti-Musashi, 1/200 (Abcam); rabbit anti-Dopamine transporter, 1/200 (Sigma); goat anti-Oct 4, 1/200 (R & D Systems); goat anti-human Sox2, 1/200 (R & D Systems); mouse anti-Tyrosine Hydroxylase, 1/50 (Sigma); mouse anti-O4, 1/400 (Millipore); mouse anti-S100, 1/200 (Abcam); mouse anti-Map2, 1/200 (Millipore); mouse anti-Neurofilament 200, 1/200 (Sigma); mouse anti-Nestin, 1/200 (Millipore); mouse anti-Prolyl-4-hydroxylase, 1/200 (BioSite, Sweden); mouse anti-Ki67, 1/200 (DakoCytomation, Denmark); mouse anti-EGFr (Epidermal growth factor receptor), 1/200 (Abcam); mouse anti-sarcomeric αActin, 1/200 (Sigma); mouse anti-cardiac Troponin I, 1/200 (Millipore); rabbit anti-smooth αActin, 1/200; and goat anti-Green Fluorescent Protein (GFP), 1/300 (Santa Cruz).

### Protocol for Immunofluorescent Double Labelling of Antigens in Glass Plates

The same procedure was followed as above except for quenching peroxidase. Secondary antibodies used were either Alexaflor 488 or 594 (anti Rabbit, anti Mouse or anti Goat as required; 1∶500). Hoechst stain was used to visualise nuclei.

### Protocol for Immunohistochemical Double Labelling of Embryo Sections

Sections were fixed in 4% PFA in PBS pH 7.4, for 20 min at RT. OCT was soaked off, sections washed in PBS and coverslipped. Relevant GFP-positive regions were located using a fluorescence microscope and photographed. Tissue specific phenotypic markers were then detected in adjacent slides using antibodies for sarcomeric αActin, cardiac Troponin I, and Neurofilament 200 overnight at 4°C or 1 h, at RT in 1% goat serum (GS) in TBS (1XTBS = 8 g NaCl, 0.2 g KCl, 3 g Tris Base, pH 7.4 in 1 L). Sections were washed twice in TBS, 0.1% Tween 20, 1% GS for 10 min, on a shaker followed by a wash without Tween 20. The secondary antibody, Alkaline phosphatase conjugated Goat α Mouse (1/100) was applied in 1% GS in TBS, for 30 min, at RT, on a shaker. Sections were washed three times in TBS, for 10 min, RT, on a shaker. Fast Red solution was made up according to manufacturer’s instructions (Sigma) and Alkaline Phosphatase reaction was undertaken for 30 min at RT. Pink staining indicating relevant tissues was photographed.

Coverslips were soaked off in PBS for 10 min. Slides were immersed in 0.3% H_2_O_2_, 0.3% Triton X100 in PBS for 15 min. They were washed twice in PBS for 5 min. Regions of interest were surrounded with Pap Pen. Non-specific binding of antibodies was blocked in 10% serum of the animal the 2° antibody was raised in (horse) in PBS. Sections were washed in PBS twice. Sections were again washed and the primary antibody, Goat-anti-GFP 1/300, applied overnight at 4°C (or 1 h, RT), in 1% horse serum (HS) in PBS. Sections were washed twice in PBS, 0.1% Tween 20, 1% HS for 10 min on a shaker and then in a wash omitting Tween 20. Slides were then incubated with 2° antibody, Biotin-Horse α Goat 1/400 in 1% HS in PBS for 30 min, at RT on a shaker. Washing and detection using ABC reagent (avidin-biotin-complex) was performed according to the manufacturer’s instructions (Vector Labs). Color development was performed in the presence of NiCl_2_ to achieve a black rather than brown precipitate. Slides were stored in 0.1% azide in PBS at 4°C. Slides were then examined under a microscope for black GFP-positive cells and photographed.

### Lentiviral Labelling of Cells with Green Fluorescence Protein (GFP)

293FT packaging cells (Invitrogen) were grown and transfected with Emerald Green Fluorescent Protein (EmGFP) construct (Vivid Colors™ pLenti6.3/V5-GW/EmGFP Expression Control Vector, Invitrogen Cat. No.V370-06) using Lipofectamine 2000 (Invitrogen) solution, according to the ViraPower Lentiviral Expression systems manual (Invitrogen). 3 µg of the plasmid DNA and 9 µg of the packaging mix (mixture of five plasmids) were used for transfection of 293 cells in one 100 mm dish. Briefly, the medium was replaced the next day and the cells were then grown for 48–72 h to generate lentivirus. The harvested supernatant was centrifuged at 3000 rpm for 20 min at 4°C, filtered through a sterile 0.45 µm low protein binding filter (Sarstedt). The virus was then concentrated in sterile SW28 ultracentrifuge tubes by ultracentrifugation (Beckman Optima™ LE-80K ultracentrifuge) equipped with a SW-28 rotor at 23,000 rpm for 1.5 h at 4°C. The pellet was resuspended in 200 µl of DMEM and aliquots of the concentrated virus were stored at −80°C. Neural stem cells (5×10^4^ cells/well) were then transduced in 24-well plates by adding 10 µl concentrated virus/well and the cells were incubated for 48 h at 37°C in 5% CO_2._ After puromycin selection (2 g/mL) for 72 h, the cells were passaged and washed several times with proprietary medium. After several passages cells were ready for transplant.

### Microarrays

#### Preparation of RNA

Qiazol mixture (Qiagen) was shaken vigorously and allowed to incubate upon the adherent cells for 2–3 min at RT before vigorous aspiration. 0.2 mL Chloroform was added to 1 mL Qiazol. The mixture was aspirated vigorously and centrifuged at 12000 g for 15 min at 4°C. The aqueous phase was collected and transferred to a new tube. 0.5 mL of isopropanol was added and the mixture incubated for 10 min at RT. Then it was centrifuged (12000 g, 15 min, 4°C) after which the supernatant was discarded carefully. The pellet was washed with 1 mL 75% ethanol. Again the mixture was centrifuged (12000 g, 15 min, 4°C) and the supernatant discarded carefully. The pellet was briefly air dried (5–10 min until pellet appeared gelatinous). It was resuspended in prewarmed (60°C) RNA Secure reagent (Ambion) and incubated for 10 min at 60°C.

#### Labelling and hybridisation

These experiments were performed at the Norwegian Microarray Consortium in Oslo, Norway. Quality control of samples: The RNA samples were delivered to NMC Oslo and kept in −80°C freezer until use. To measure the concentration of each sample a NanoDrop spectrophotometer was used. A Bioanalyzer (Experion, Bio-Rad) analysis was performed in order to check if the samples were intact after transport to the facility. Total RNA was used for amplification and labelling. The quality of the product cRNA was assessed using a Bioanalyzer. 1.5 µg of biotin labeled cRNA was used to hybridise onto Illumina HumanWG-6 v3 Expression BeadChips.

Microarray data from our lab are MIAME compliant and the following link has been created to allow review of the data in Gene Expression Omnibus [25] (record GEO accession number GSE41470): http://www.ncbi.nlm.nih.gov/geo/query/acc.cgi?acc=GSE41470.

#### Array normalisation for comparison with published array data

The microarrays were normalized using quantile normalization [26], where the first six arrays (three SVZ samples and three HPC samples; done together on the same platform and day) were used as reference samples. The values in these arrays have been left unchanged. Then the other arrays have been normalized according to the first six the following way: The gene ranked as number one (having highest value) in array X, is given the median value of the six genes ranked as number one in each of the arrays in the reference set. The same is done to the gene ranked as number 2 in array X. And so on, for all genes, and all arrays (not in the reference set).

J-Express Fold Change tool has been used in an attempt to relate various ‘adult stem cell’ types. Tabular expression comparisons were made of the number of genes differing greater than 3-fold between our various cultures as well as other stem cell culture types from published arrays (Table S2 ). The data from all arrays were quantile normalized and their graphic distribution of expression levels ‘smoothed’ by a statistician such that Array number 1 with two groups: three SVZ and three HPC expression arrays has been used as the standard (see above). Only genes common to all arrays were included. The total number of genes in this analysis was 7264.

#### Arrays used in the comparison

The first six arrays are this lab’s and are of three hippocampus (HPC) and three subventricular zone (SVZ) –derived stem cell samples. The second six arrays (also ours) are grey matter (GM) and white matter (WM) –derived stem cells each related to two conditions: Grey matter tissue (GMt), Grey matter differentiated cells (GMd) and similarly, WMt and WMd. The platform for these 12 was Illumina Human-6 V3 Bead Chip. The first six are the reference samples to compare all the rest with.

Then there are six arrays of mesenchymal stem cells on Affimetrix H6-U133A [27] Array Express E-MEXP-214 and 215.).

There are five human subventricular zone-derived neurosphere samples on ABI Human Genome Survey microarray version 2 [28] Gene Expression Omnibus GSE31262).

There are 12 arrays of Human olfactory neural stem cells on Illumina Bead Array Ref 8V2 [29].

We have a further 12 arrays on Illumina WG-6 V3 Bead Chip. These are similar to the first 12 arrays. The samples are: Brain stem cells grown adherently, Brain SVZ stem cells grown as neurospheres, Tumour (Glioblastoma) stem cells grown adherently, and these same samples grown as neurospheres (three samples of each type).

#### Statistical comparison of SVZ and HPC-derived cultures

To test whether the hippocampus (HPC) and subventricular (SVZ) expression values are statistically different from each other (more than you can expect by chance) we first used the method *locfdr* (local false discovery rate, [30]). Later, the method *globaltest* [31] was applied to reappraise statistical differences over four brain stem cell sources (see below).

Subsequently a further 12 arrays on Illumina WG-6 V3 Bead Chip were processed. One platform carried triplicate cultures from three humans for four cell sources (HPC, SVZ, GM and WM) all grown at the same time in identical conditions to the same number of cells and processed together. This was intended to test the hypothesis that these cell populations were identical. The data in these arrays was subjected to *globaltest* (mentioned above) and other analyses.

### Western Blot

#### Preparation of protein extract and western analysis

Adherent cells (undifferentiated and ‘differentiated’) were washed in PBS, scraped and pelleted at 300×g for 5 min. The cells were homogenized by triturating in Cell Extraction Buffer (Mammalian cell extraction kit K269-500, Biovision). The homogenates were then vortexed for few seconds, incubated on ice for 10 minutes and finally centrifuged at max speed for 1 min through QIAshredder (Qiagen). The supernatants from each sample were collected, and the amount of total protein was determined using the BCA protein assay kit (Thermoscientific). 20–40 µg of whole protein extracts were mixed with the loading buffer (NuPAGE) and loaded onto a 4–12% gradient Nu-PAGE gel (Invitrogen). Protein gels were blotted onto a 0.45 µm PVDF membrane. The membrane was blocked with 5% skimmed milk in TBS/0.1% Tween 20 (TBST) and probed with Oct-4 (9B7) Mouse mAb (#4286 Cell Signaling; 1/1000), Tyrosine Hydroxylase Rabbit Antibody (#2792 Cell Signaling; 1/1000), or β-Actin Rabbit Antibody (#4967 Cell Signaling; 1/1000) in TBST and 5% skimmed milk. Secondary antibody was HRP-conjugated anti-rabbit IgG (1/10000 in 5% skimmed milk in TBST) or anti-mouse IgG (1/10 000 in 5% skimmed milk in TBST). The blots were developed using Lumiglo Reserve CL Substrate kit, and detected by Agilent Molecular Imaging System (Unilabs).

### Proteomic Studies

#### Cell culture and SILAC

The ‘SILAC’ (Stable Isotope Labeling by Amino acids in Cell culture) cell culture medium (D-MEM/F-12), deficient in Arginine and Lysine, was custom made (Invitrogen). NaHCO_3_ (29 µM, Sigma) was added and pH set to 7.4. Heavy amino acids [13C6]-L-Lysine and [13C6]-L-Arginine and also the corresponding light amino acids were obtained from Invitrogen. All cells were grown adherently: SVZ cells were grown on light medium (containing light amino acids (100 mg/L), Heparin (2.5 mg/mL, LEO Pharma AS), Hepes (10 mM, LONZA), P/S (100 U/mL, LONZA), B-27 without Vitamin A (2% (v/v), GIBCO), TGF-α (20 ng/mL, R&D Systems), bFGF (10 ng/mL, R & D Systems) and dialyzed fetal bovine serum (1% (v/v), Invitrogen). HPC cells grown on heavy medium (containing heavy amino acids (100 mg/L), Heparin (2.5 mg/mL, LEO Pharma AS), Hepes (10 mM, LONZA), P/S (100 U/mL, LONZA), B-27 without Vitamin A (2% (v/v), GIBCO), TGF-α (20 ng/mL, R&D Systems), bFGF (10 ng/mL, R&D Systems) and dialyzed fetal bovine serum (1% (v/v), Invitrogen). All cells were passaged three times to attain complete labelling.

#### Protein extraction and electrophoresis

Cells were harvested and counted. Pre-heated lysis buffer (SDS, 1% Na_3_VO_4_, 2 mM Tris-HCl (pH 7.4), 10 mM NaF was added to one million cells and the cells were incubated at 95°C for 5 min. The cells were then homogenized and sonicated. Protein concentration determination was conducted using the DC Protein Assay (Bio Rad, Hercules, CA, USA). Proteins from the three biological replicates were used. SVZ had light amino acids and HPC had heavy amino acids. Equal amounts of the lysates, from heavy and light samples, to a total amount of 50 µg were mixed and Clear PAGE sample buffer (25%, CBS Scientific) was added. The samples were heated at 70°C for 10 min. The proteins were separated with SDS-Reducing Running Buffer (CBS Scientific) on a ClearPAGE gel 12 well, gradient gel 4–12% (CBS Scientific) at 150 V for 60 min and then Coomassie G-250 stained.

#### Nano-LC/LTQ-Orbitrap mass spectrometry

The Coomassie G-250 stained single gel lanes of three biological replicates were excised for in-gel digestion with 0.1 µg of trypsin (Promega, Madison, WI, USA) in 20 µL 25 mM ammonium bicarbonate, pH 7.8 at 37°C for 16 h. The dried peptides were dissolved in 10 µL 1% formic acid in water and 5 µL were injected into an Ultimate 3000 nano-LC system (Dionex, Sunnyvale CA, USA) connected to a linear quadrupole ion trap-orbitrap (LTQ-Orbitrap XL) mass spectrometer (ThermoScientific, Bremen, Germany) equipped with a nanoelectrospray ion source. An Acclaim PepMap 100 column (C18, 3 µm, 100 Å) (Dionex) with a capillary of 12 cm bed length was used for separation by liquid chromatography. A flow rate of 300 nL/min was employed with a solvent gradient of 7% B to 40% B in 87 min, then 40–80% B in 8 min and subsequently from 40 to 80% B in 8 min. Solvent A was 0.1% formic acid, whereas aqueous 90% acetonitrile in 0.1% formic acid was used as solvent B.

The mass spectrometer was operated in the data-dependent mode to automatically switch between Orbitrap-MS and LTQ-MS/MS acquisition. Survey full scan MS spectra (from m/z 300 to 2,000) were acquired in the Orbitrap with resolution R = 60,000 at m/z 400 (after accumulation to a target of 1,000,000 charges in the LTQ). The method used allowed sequential isolation of the most intense ions, up to six, depending on signal intensity, for fragmentation on the linear ion trap using collisional induced dissociation (CID) at a target value of 100,000 charges.

For accurate mass measurements the lock mass option was enabled in MS mode and the polydimethylcyclosiloxane (PCM) ions generated in the electrospray process from ambient air were used for internal recalibration during the analysis [32]. Target ions already selected for MS/MS were dynamically excluded for 90 seconds. Other instrument parameters were previously described [33].

#### Data analysis

Instrument raw data was processed and quantified using MaxQuant [34]. For processing, the top 6 MS/MS peaks per 100 Da were used to generate msn-files. These msn-files were searched against the human IPI database (v. 3.62, 83685 sequences) containing both reversed sequences and contaminants using an in-house version of the Mascot search engine (v.2.2.1). A mass tolerance of 0.5 Da was used for MS/MS fragments. Trypsin was used as protease allowing up to one missed cleavage and peptide charge 2+ and 3+. As variable modifications oxidation (met) and N-acetyl (protein) were allowed. At least two peptides, with one of them a unique peptide, were required for protein identification and as well a False Discovery Rate of 1% was applied. For protein quantification, at least two ratio counts were required and razor peptides were included in the calculation of protein ratios.

To assess the regulated proteins, the Grubbs test for outliers was applied. First the distribution of protein ratios were iteratively checked for outliers using the Grubbs test, and every outlier removed. The remaining population’s mean and standard deviation (SD) (calculated in log space) was used to describe the un-regulated population. All proteins outside 1.96SD of this population were considered to be regulated.

### G-banding and Karyotyping

The cells were cultured in the optimal medium for adherent culture. Cells from one, two, or three passages of the individual samples were cultured for 2–5 days before they were harvested as described by Mandahl and co-workers [35]. Chromosome preparations were G-banded using Wright stain and karyotyped according to the ISCN(2009) [36] guidelines.

### Microscopy

Cells and embryos were examined and photographed using a Motic 31 inverted fluorescence microscope with Nikon D40X digital camera, a Zeiss Axiovert S100 fluorescence microscope with Princeton Instruments RTE/CCD1300 camera and Axiovision software, and a Zeiss Axioskop 2 light microscope fitted with an Axiocam color camera and Axiovision software. Images were labelled using Adobe Photoshop.

### Cell Transplant in Chick Embryos

Human neural stem cells were enzymatically dissociated and labelled with Lenti-GFP (see above) or CMFDA (Molecular Probes) according to the manufacturer’s instructions. Fertile white Leghorn chicken eggs were incubated at 37°C for approximately 20–24 h (stage 4–6). Shells above the air space region were removed and dissociated stem cells were injected into the region of the primitive streak using a micromanipulator (PV 830 Pneumatic picopump, Germany) and a drawn glass micropipette. Two µL of cells (1,000 cells/µl) were injected. Eggs were then resealed and opened for processing 2–5 days later. Embryos were fixed in 4% PFA and cryoprotected in 20% sucrose in PBS before being frozen in OCT (Sakura, Netherlands).

### Transplant into Mouse Brains

All animal procedures were approved by the National Animal Research Authority (1094/2008). C.B.-17 severe combined immunodeficient mice (SCID) (7–9 weeks old, Taconic, Ejby, Denmark) were transplanted according to previously published protocol [9]. A 2 µL suspension containing 100 000 cells/µL, was injected into the right striatum just below the corpus callosum (AP 0 mm, RV 2 mm, ML 2 mm) using a cannula (Plastic One, Roanoke, VA) attached to a Hamilton syringe (Hamilton Bonaduz, Bonaduz, Switzerland). Mice were observed for up to six months before sacrifice by transcardial perfusion with 4% paraformaldehyde.

## Results

### A New Culturing Protocol

Using established culture protocols for growing neurospheres it is possible to passage stem/progenitor cells from the adult human brain only a very few times and it is most often impossible to expand cell numbers. In order to try to overcome these difficulties we systematically tested modification of the standard ‘VML’ neurosphere culture medium: (DMEM/F12 supplemented with B27, hepes, heparin, penicillin, streptomycin, bFGF and EGF –see **Materials and Methods**) by adding or changing concentration of various test ingredients, singly or in combination. Cell culture characteristics were monitored twice weekly. Neurosphere cultures were passaged when spheres reached 12–15 cells in diameter. Adherent cultures were passaged at 70–80% confluence, i.e. prior to contact inhibition. The total number of cells was determined at each passage and monitored in order to determine the cultures with the greatest generative ability.

**Figure 1:**
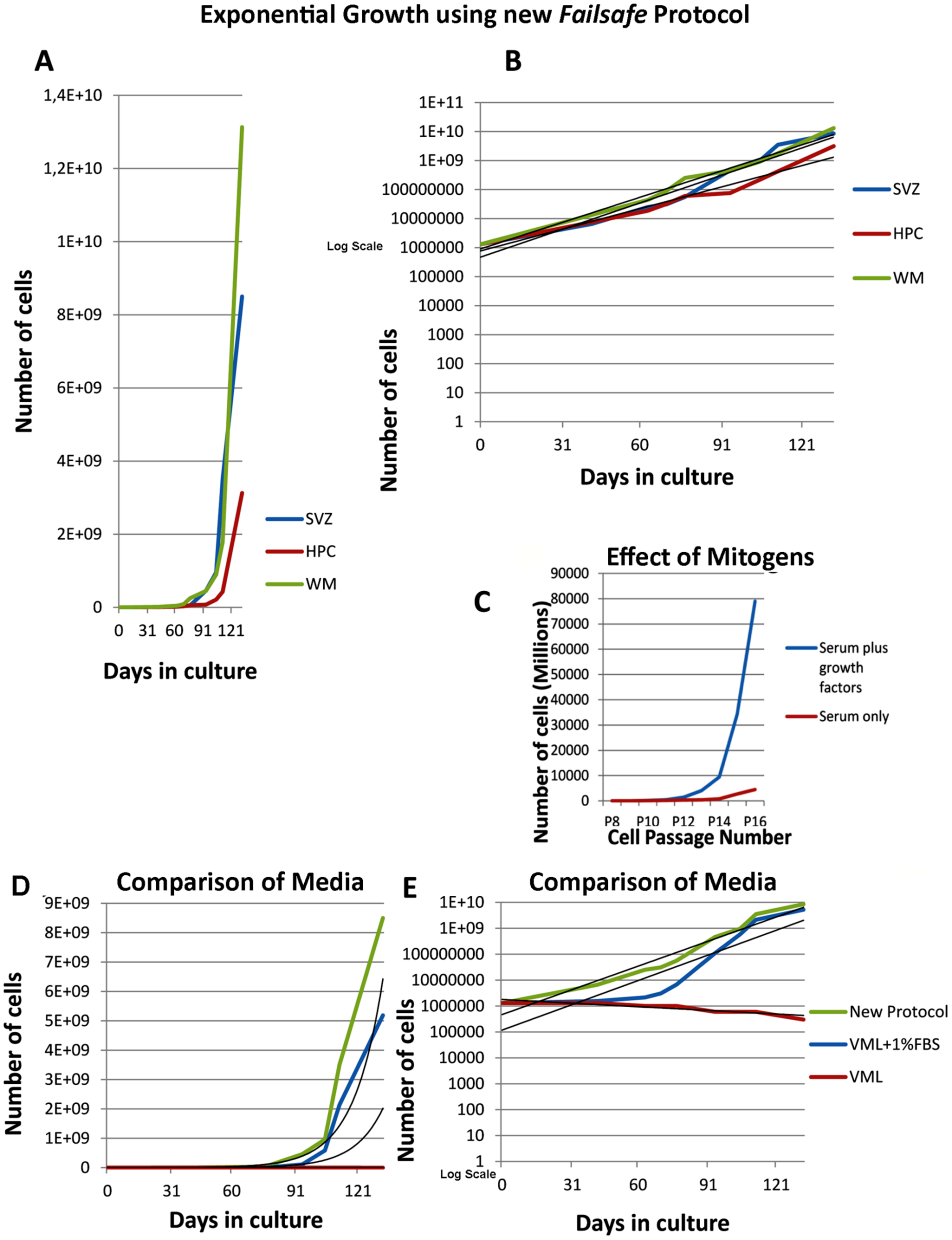
Growth of human brain-derived stem/progenitor cells. **A.** **and B.** Early expansion of cells using the optimal culturing medium. All cultures soon acquire exponential growth even from small biopsies. **B.** presents **A.** using a Log Scale. Black lines are ‘Trend lines’ generated by *Excel* Software. **C.** Clearly serum (1%) supplied an essential nutrient but serum in the absence of mitogens (FGF2 and TGFa or EGF) failed to induce rapid growth. **D. and E.** Comparison of media. **E.** presents **D.** using a Log Scale. Black lines are ‘Trend lines’ generated by *Excel* Software.

In four years we have grown more than 320 tumor cell lines and 50 lines from 34 normal patients. The human patient sources for the cell populations from which the data are derived ranged between 22 and 53 years of age and were of both sexes. The factors that most significantly affected expansion of the normal cells were FBS and mitogens EGF and bFGF (Figure 1). With the standard VML neurosphere medium it was only possible to passage the cells 2–3 times, as we and others have reported earlier, and it was virtually impossible to expand the number of cells. When 1% FBS was added the cells adhered to the dish and grew as adherent monolayers, but could be passaged many times with expansion of cell numbers with each subsequent passage (Figure 1). Removal of growth factors from the medium resulted in severe reduction of expansion (Figure 1C). Substitution of EGF with TGFα which activates the same receptor, significantly further increased cell numbers (Fig. 1D, E), presumably because this growth factor is readily released and recycled [37].

**Figure 2:**
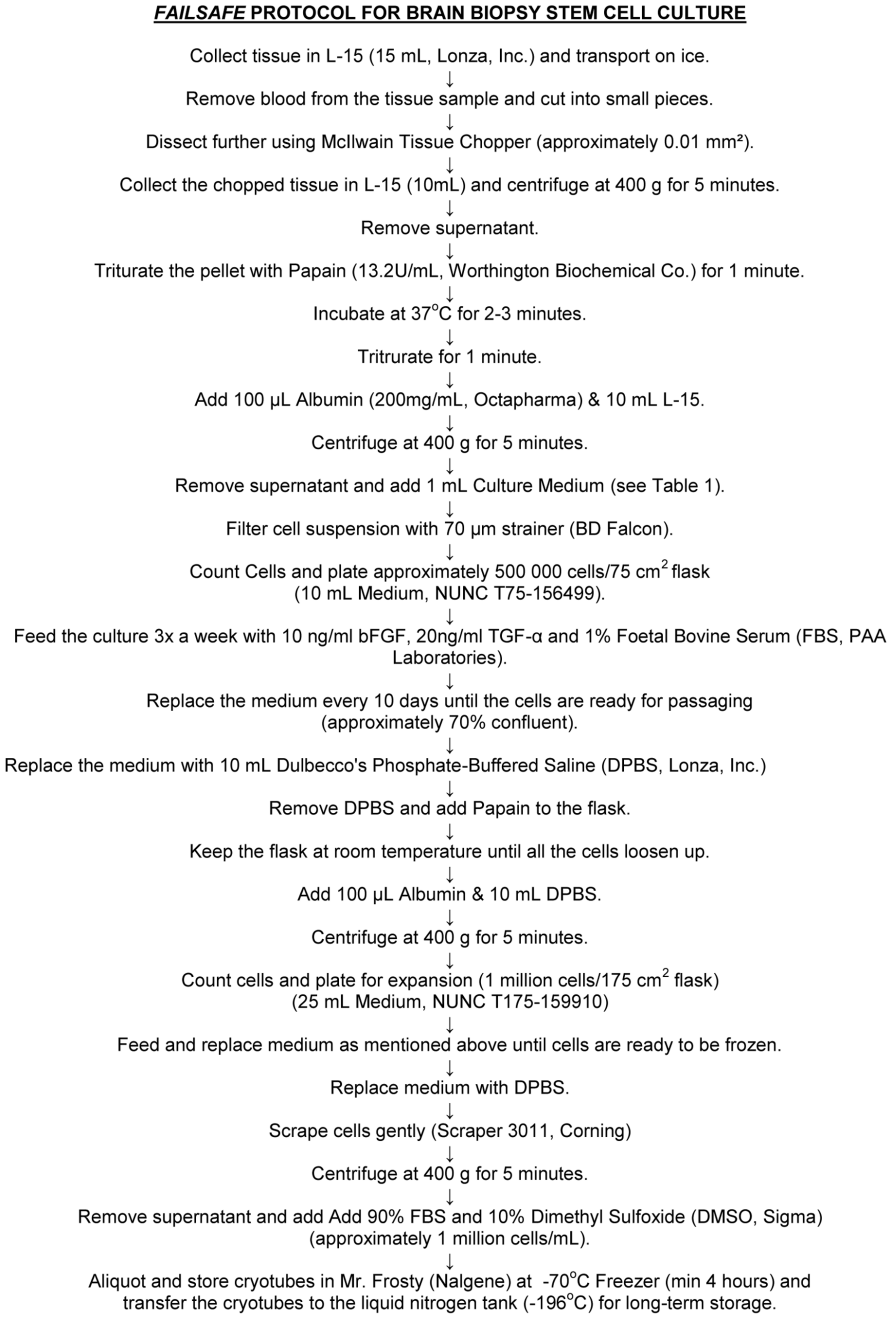
*Failsafe* protocol for brain biopsy stem cell culture.

The optimal culture medium was DMEM/F12 with 10 ng/mL bFGF, 20 ng/mL TGFα, 2.5 µg/mL heparin, 2% B27 (without retinoic acid), 10 mM hepes, 1% Pen/Strep and 1% FBS (Figure 2, Table 1). Cell cultures were kept in a 37°C incubator with 5% CO_2_.

**Table 1:** Constituents of the optimal growth medium.

Medium Components	Final Concentration
Hepes Buffer (Lonza)	10 mM
Pen/Strep (Lonza)	100 U/mL
B27 without Vit A (Gibco)	2 mL (50X)/100 mL Culture Medium
bFGF (R&D Systems)	10 ng/mL
TGF-α (R&D Systems)	20 ng/mL
Heparin (Leo Pharma AS)	2.5 µg/mL
FBS (PAA Laboratories)	1%
DMEM/F12 (Life Technologies, Inc)	1X

**Figure 3:**
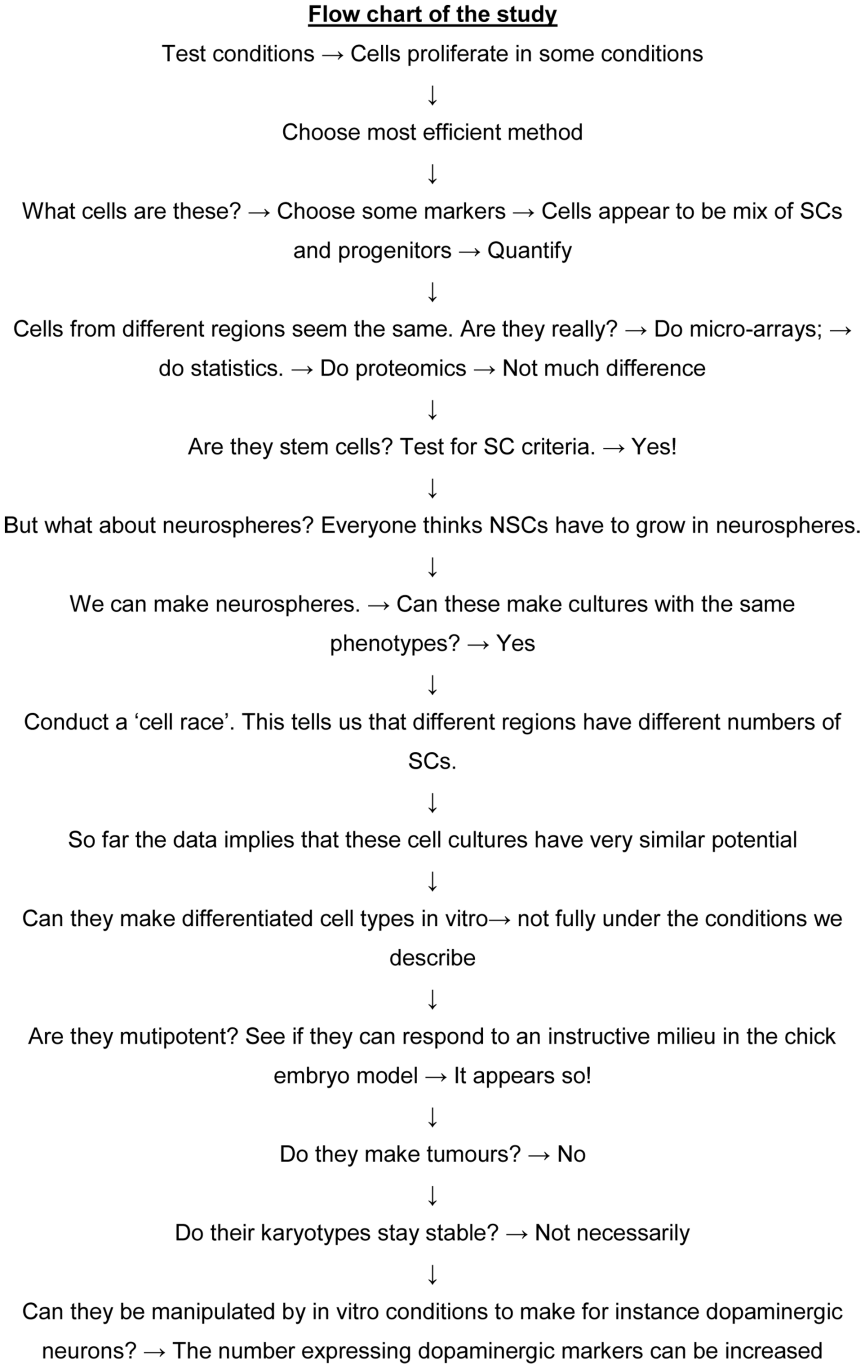
Logical rationale for collecting the data presented.

**Figure 4:**
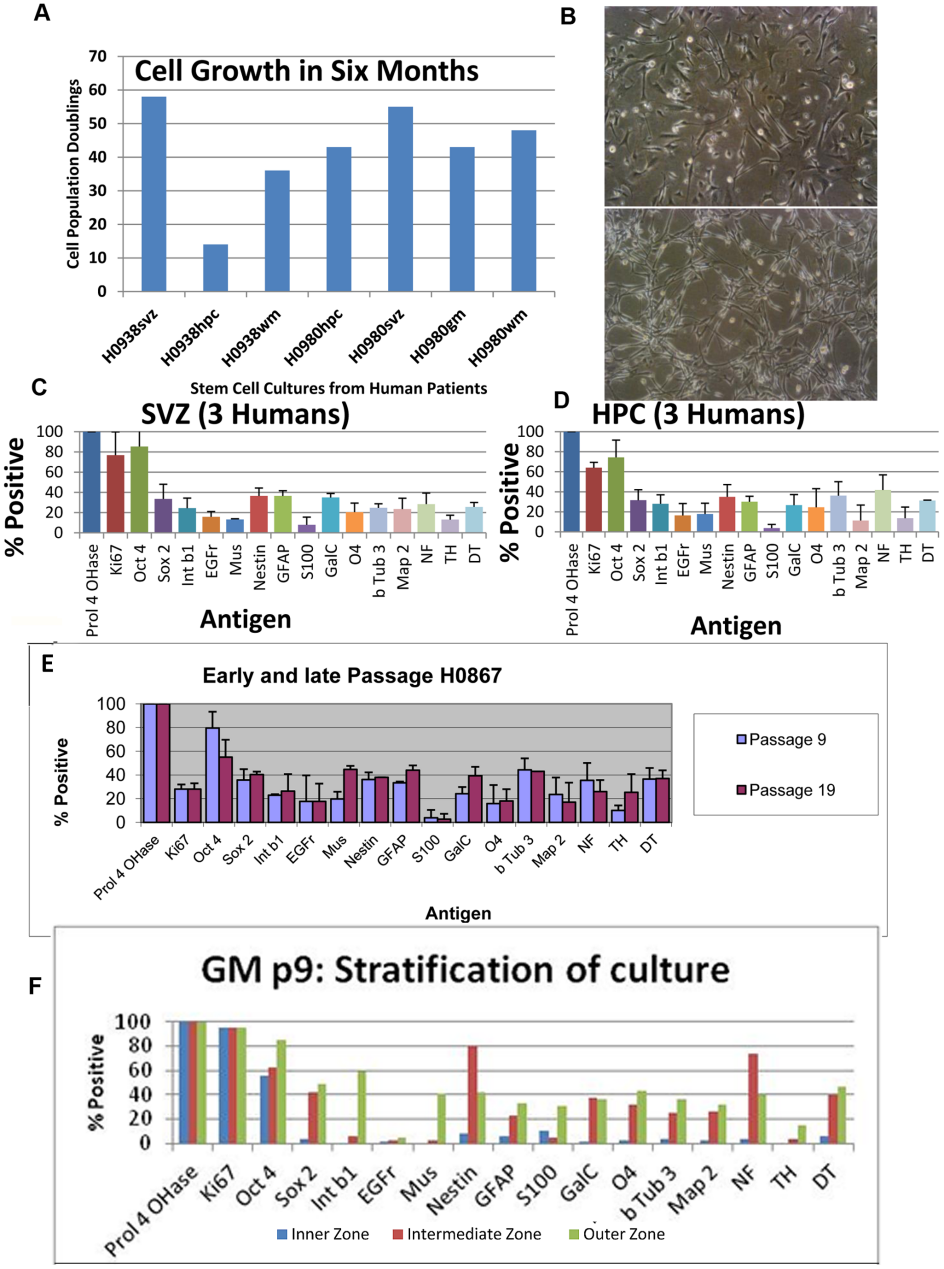
Proliferation and phenotype of brain stem cells. **A.** Average doubling time was 6.5 days. 50 doublings is >10^14^. This is a yield of 10^18^ from a small biopsy. There are 10^14^ cells in a human. **B.** Morphology of brain-derived stem cells growing adherently. **C and D.** Cultures derived from Hippocampus appeared identical to those from Subventricular zone. (Bars: +SD, n = 3). Markers of: +ve control, Prol4OHase; dividing cells, Ki67; stem cells, Oct 4, Sox2, Intβ1, EGFr, Mus, Nestin; glia, GFAP, S100; neurons, βTub3, Map2, NF; oligodendrocytes, O4, GalC; dopaminergic cells, TH, DT. **E.** Cultures maintained phenotype through many passages. (Bars: +SD, n = 3). **F.** **Stratification of cultures (Grey Matter p9).** Cultures could be arbitrarily divided into zones based on the appearance of the cells in these zones. Immunophenotype confirmed that the differences in shape also reflected differences in phenotype and suggest that this stratification in some way reflects the dynamics involved in tissue organization (See also **Figure S1. Phenotype of brain stem cell cultures**).

At this point we began an experimental scheme designed to answer the questions outlined in the Introduction, paragraph 4 (Figure 3). Under the *Failsafe* conditions cells expanded exponentially (Figure 1A, B). We tested these culturing conditions on cells harvested from 34 patients and found that cells propagated robustly in all cases. We routinely expand cells for more than 10 passages. In seven cases we expanded cell lines for more than six months with average doubling times of 6.5 days (Figure 4A). 50 doublings is >10^14^. This is a yield of 10^18^ from a small biopsy. There are 10^14^ cells in a human.

Glioblastoma stem cells, which usually grow exponentially as spheres, doubled at about twice the rate with this protocol. Additionally cells from all glioblastoma patients grew in these conditions, as compared to only four out of five when grown as spheres. Cells from low grade gliomas could also be passaged and expanded in numbers when using this medium.

### Phenotype of Progenitors

**Figure 5:**
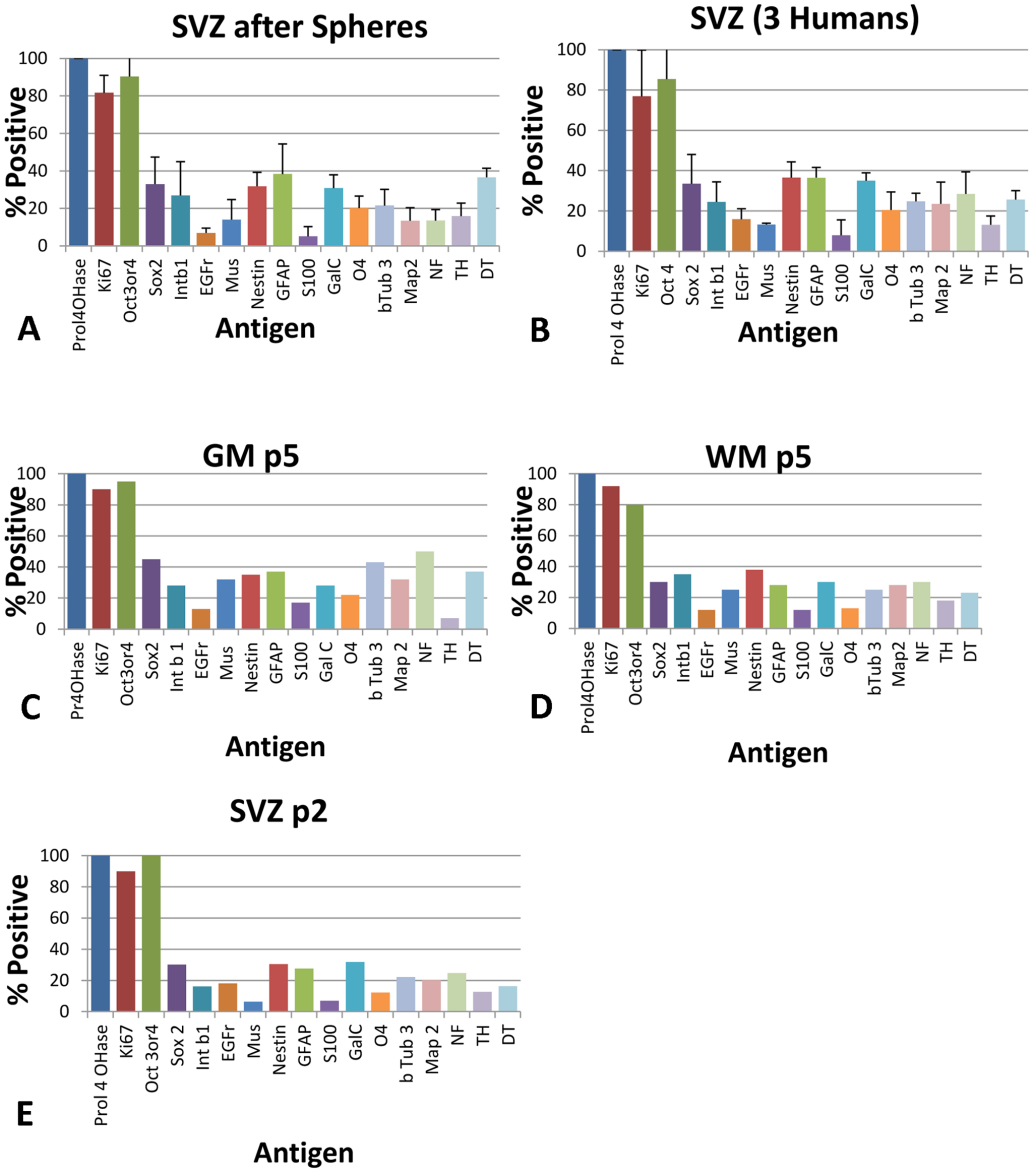
Sphere-derived and early passages. **A and B**: Comparison of three SVZ-derived cultures after being grown as neurospheres with three grown consistently adherently. (n = 3 for each; Error bars: +SD). **C, D, E**: Examples of early passaged cultures (n = 1).

Next the immunological phenotype of these cultures was assessed. These analyses were done in plastic multiwell plates with no substrate (substrate clearly influences phenotype) using chemical detection (plastic interferes with immunofluorescence photography); and assessed as convenient. (This analysis was done on multiple cultures from multiple biopsies coming at unpredictable times.) Chemical detection is very sensitive and does not fade with time or light. As well it takes a good while to achieve sufficient cell numbers for expansion, storage and phenotype assessment. 15 antigens were chosen. It was expected that cultures would not be homogeneous as has already been shown in other neural progenitor populations so putative markers were chosen to assay presence of stem cells: OCT 4, SOX 2, integrin-β1, EGF receptor (EGFr); neural stem cells: musashi, nestin, neurons: β-tubulin 3(βTub3, TUBB3), MAP2, neurofilament 200 (NF), glia: GFAP, S100, oligodendrocytes: O4, GAL C, dividing cells: KI67 and a positive control ubiquitous antigen (a subunit of prolyl-4-hydroxylase, Prol4OHase). As well cultures were assessed for the presence of dopaminergic phenotype: tyrosine hydroxylase (TH) and dopamine transporter (DT) as attempts to differentiate in this direction were envisaged for later experiments. Cultures were assayed as well for non-neural tissue cell types using antibodies to cardiac Troponin I, smooth muscle αActin and striated muscle αActin. Cultures were negative for these non neural markers. Cultures were assessed for percentage positive cells in triplicate from both Hippocampus (HPC) and Subventricular Zone (SVZ) of three humans (Figure 4B and C) allowing assessment of variation of cultures. Cultures were then obtained from two humans for Grey Matter (GM) and White Matter (WM). Notably, these cultures conformed to the range of expression found for SVZ and HPC and cells looked similar. As well slow attaching cells (sometimes these are assumed to possess different developmental potential) were assessed for the same phenotype markers and fell within the range already seen. Figure 5 depicts examples of SVZ cultures grown adherently after growth as spheres, cells grown consistently adherently and also of early passage cultures.

It would have been good to use serum-free neurospheres as a control cell type but the nature of human adult neurosphere culture is such that one could not possibly obtain enough cells to do the multiwell analysis that we have done here. Most papers have reported sphere phenotype after plating on substrate protein of some sort and adding serum whilst withdrawing mitogens (in other words: ‘differentiating’ conditions). So it is very difficult to compare their results. The problem is exacerbated by the difficulty of separating or permeabilizing the sphere cells. With olfactory stem cells the sphere cells were enzymatically separated and then stained and counted [38]. We got 17% neurons, 79% astrocytes, 53% Nestin+ cells. In our lab we have fixed, embedded and sectioned human brain neurospheres. We got 20% neurons, 77% astrocytes, 32% oligodendrocytes, 49% nestin+, 35% sox2+ (Vik-Mo et al, unpublished). The results of these studies (mentioned above) are detailed in: **Table S1. Phenotype of brain stem cell cultures.** A figure (5A) depicts phenotype (on plastic) after being grown as spheres.

### Cell Populations Possessed Common Characteristics Despite Origin

As can be seen (Fig. 4C,D,E) significant percentages of cells expressed stem cell markers as well as neuronal, oligodendrocyte and astrocyte markers. Of particular note OCT 4 was expressed in greater than 80% of cells. Before this time OCT4 was only seen at a very low level or not at all in neurospheres from similar sources.

Cultures could be arbitrarily divided into zones based on the appearance of the cells in these zones (Figure 4F, **Figure S1. Phenotype of brain stem cell cultures.**). Cells of the inner zone were rounder and flatter, those of the middle zone were beginning to show multipolarity and those of the outer zone more filamentous, often bipolar and elongate. Immunophenotype confirmed that the differences in shape also reflected differences in phenotype and suggest that this stratification in some way reflects the dynamics involved in tissue organization (Fig. 4F). For example neuronal phenotype was strongest in the outer zone (elongate cells) whereas nestin was strongest in the intermediate zone. In all subsequent experiments whole populations of cells (from wells) were used collectively so that no bias of phenotype relating to dish region could play a part. We have provided some double immunofluorescence pictures as well. See: **Figure S2. Double staining using immunofluorescent secondary antibodies.** Oct4 co-localizes with GFAP often, sometimes with NF and TUBB3. Nestin co-localizes often with GFAP, sometimes TUBB3, sometimes O4. O4 sometimes co-localizes with TUBB3. TH can co-localize with DT. TH can co-localize with TUBB3. NF can co-localize with DT. We do not have suitable antibodies to test for other co-localizations at this time. The implication of this data is that many of the cells are early precursors that have not completed cell-type fate decisions. This is especially indicated in that most cells still express OCT 4.

**Figure 6:**
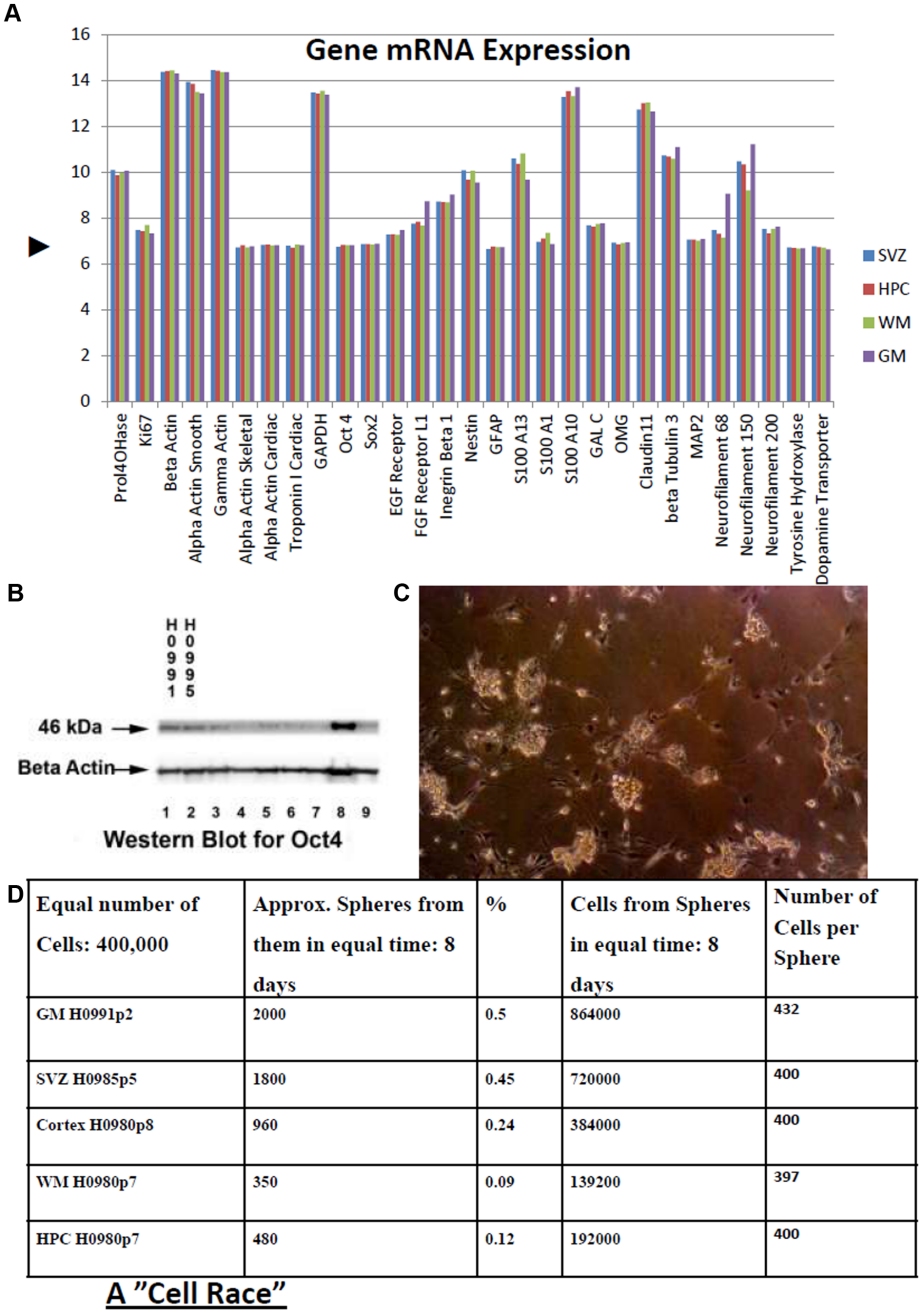
Characteristics of human brain stem cells. **A.** mRNA Phenotype of four groups of brain derived stem cells. mRNA levels in stem cell cultures (mean of three humans) comparing those derived from Subventricular Zone (SVZ), Hippocampus (HPC), White Matter (WM) and Grey Matter (GM). These are some common genes some of which were already assessed using immunohistochemical techniques as we have described above. Data are from the same microarray platform and were processed together. Ordinate axis units are mean probe strength (Log_2_) recorded for the genes specified on the co-ordinate axis. Microarray data were ‘quantile normalised’. **▸**: Basal level of transcription. Markers: Housekeeping: Prolyl-4-hydroxylase, β-actin, γ-actin, *GAPDH*. Dividing cells: *KI67*. Stem cells: *OCT 4*, *SOX2*, *EGF* receptor, integrin-β1, nestin. Neurons: β-tubulin3, *MAP2*, neurofilament. Glia: *GFAP, S100*. Oligodendrocytes: *OMG*, claudin11, *GALC*. Smooth muscle: α-actin smooth. Skeletal muscle: α-actin skeletal. Cardiac muscle: α-actin cardiac, cardiac troponin I. Dopaminergic cells: Tyrosine hydroxylase, dopamine transporter. **B.** Western blot confirmed robust expression of stem cell ‘pluripotency’ marker OCT 4 (POU5f1). 40 µg total protein was run on each lane. Lanes 1 and 2: normal samples: 1, HPC, 2, SVZ; Lanes 3–9: tumor stem cells. **C.** Cells (grown adherently) and then subsequently plated in neurosphere-forming conditions. **D.** From Table in D, it can be seen that though the sphere generating cells from the different sources varied in density, the cells generated from them had similar growth ability (number of cells per sphere).

Next the cultures were assessed for ability to generate neurospheres as neurosphere-generating ability is believed by many to indicate the presence of neural stem cells. As the cells were growing adherently (similarly to olfactory stem cell cultures) [38], [39], [40] the same method of growing olfactory neurospheres was attempted where cultures were grown on 1 µg/cm^2^ poly-L-lysine, 50 ng/mL EGF, 25 ng/mL bFGF, ITS, P/S. This method produced neurospheres after many days, however an optimal method was found to be 1 µg/cm^2^ poly-L-lysine, 2% vol/vol B27 without RA, 20 ng/mL EGF, 10 ng/mL bFGF, 0.5 µg/mL heparin, 10 mM hepes, 30 µg/mL bovine pituitary extract (BPE) and 1% P/S. Under these conditions abundant neurospheres appeared in two days starting as irregular clusters (Figure 6C, **Figure S3. Neurospheres in suspension culture**) that eventually detached and floated off after several more days. Next the ability of cultures derived from the different regions was tested for sphere-forming capacity (Figure 5A and 6C, D). Neurospheres were then tested for ability to reconstitute adherent cultures with similar immuno-phenotypes as had generated them. The characteristics of these cultures were within the range for the tested markers as before (Figure 5A). As well, the ability of different region-derived cultures was tested for sphere-forming capacity followed by cell generating capacity (Table in Figure 6D). It can be seen that though the sphere generating cells from the different sources varied in density, the cells generated from them had similar growth ability (cells/spheres).

### Stem Cell Phenotype Confirmed

Western blot was used to confirm some of the markers: βIII tubulin, GFAP, as well as OCT 4. The robust expression of OCT 4 at the known size of 46 kDaltons confirmed the stem cell nature of these cultures (Figure 6B).

Microarrays were obtained for a number of cultures allowing assessment of RNA expression for relevant markers as well as to measure relatedness of the cultures from different cellular regions. Most genes detected with immunochemistry were shown to be actively expressed at RNA level albeit some at low level (Figure 6A). This figure presents mRNA levels in stem cell cultures (mean of three humans) comparing those derived from Subventricular Zone (SVZ), Hippocampus (HPC), White Matter (WM) and Grey Matter (GM). These are some common genes some of which were already assessed using immunohistochemical techniques as we have described above. Data are from the same microarray platform and were processed together. Ordinate axis units are mean probe strength (Log_2_) recorded for the genes specified on the co-ordinate axis. Microarray data were ‘quantile normalised’ by the Norwegian Micro-array Consortium. **▸** depicts **‘**Basal level of transcription’ ie. The level at which signal above background signal becomes statistically significant.

**Figure 7:**
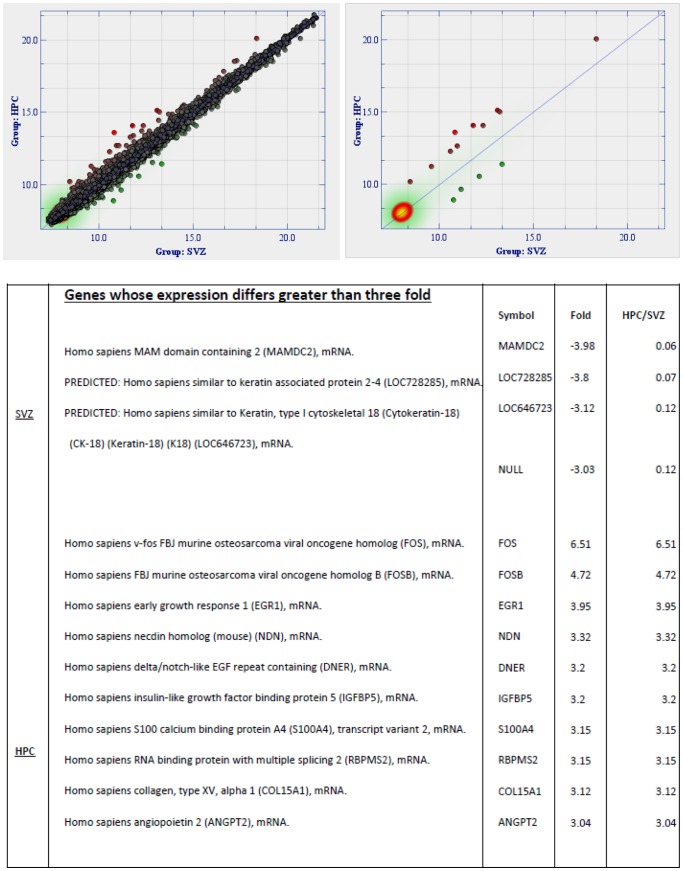
SVZ compared to HPC. Graphic output from the ‘Fold Change’ tool of J-Express software for microarray analysis comparing a group of three hippocampus-derived stem cell lines with those derived from the same three patients’ subventricular zone-derived stem cells. Left side: Differences in 37880 individual gene expression levels (distance from the diagonal) are indicated by the colored dots. Genes differing by three or greater fold (14 only) between the two groups are indicated on the right side. **Table** List of genes differing more than 3-fold between three SVZ-derived and three HPC-derived stem cell lines.

As well the relatedness of the cultures could be measured. Looking for differences in expression of greater than three fold indicated for example that only 14 genes out of a total of 37880 differed that much in expression between those derived from Subventricular Zone (SVZ) and those derived from Hippocampus (HPC) (Figure 7).

Other cultures have been tested and the relatedness by this criterion is indicated in: **Table S2**. The table includes published array data. All arrays have been ‘normalised’ by a statistician. The methodology used is described (**Materials and Methods**).

The *locfdr* method [30]was used to investigate whether the HPC and SVZ-derived cultures have expression values that are really different from each other (see **Material and Methods**).

**Figure 8:**
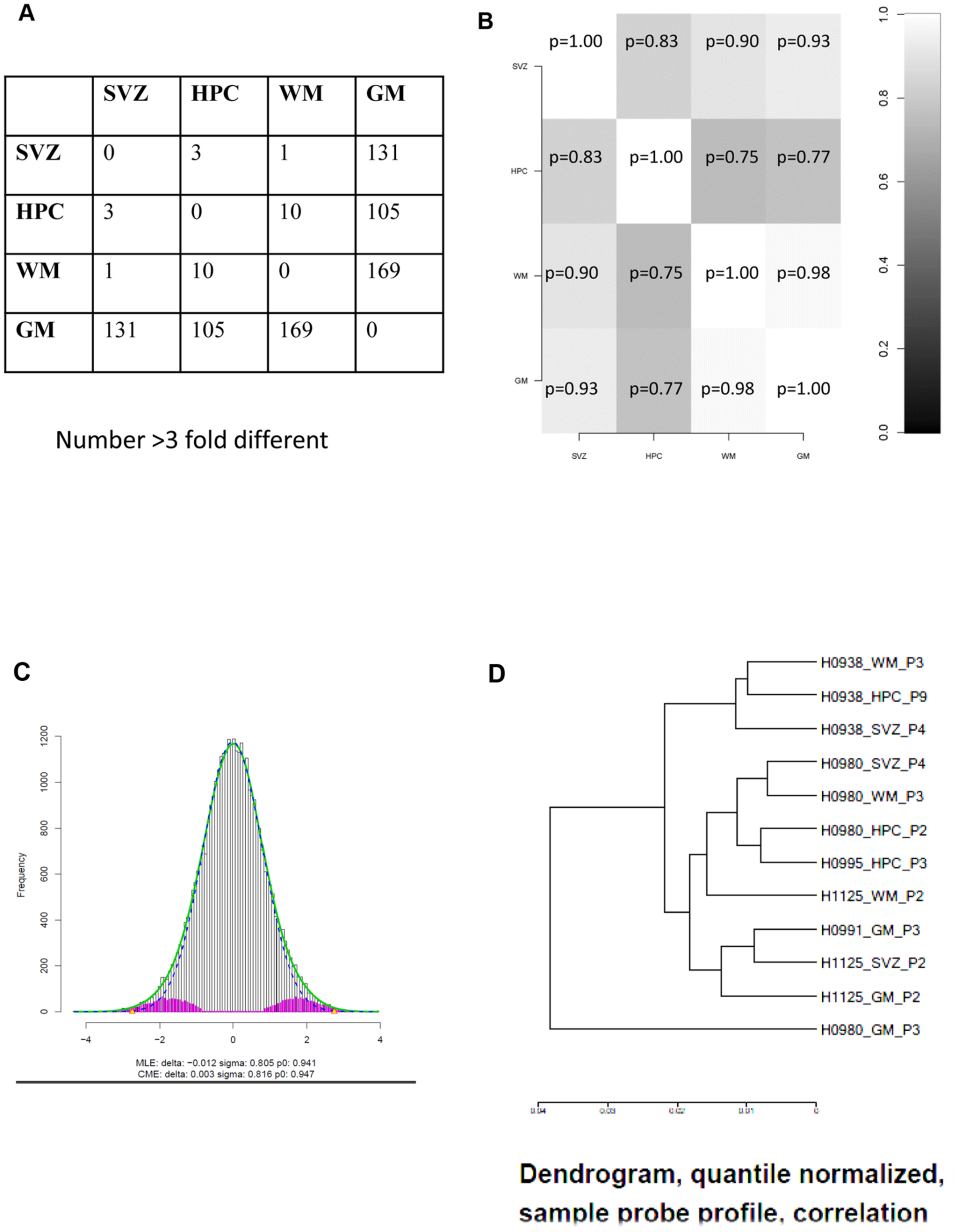
Are stem cell sources different based on microarray analysis. Relatedness of cultures from 3 humans/4 sources/1 platform. **A.** Two way table showing number of genes (mean; n = 3) differing by more than three fold. Total number of genes in this comparison: 34694. **B.** ‘Globaltest’ analysis revealing probability for the null hypothesis after testing differences between cell culture groups (n = 3). **C**. Histogram: the white bars show a test statistic for a paired *t*-test of HPC vs SVZ (individual cultures from each human have been paired). The blue line shows what one would expect if there were no real differences (just random, due to technical noise etc). The pink bars are what the method estimates as real differences calculated as the difference between the white bars and the blue line). The method estimates that around 6% of the genes are different between SVZ and HPC. **D.** The similarity between the expression profiles is determined by hierarchical cluster analysis and shown as a dendrogram. The length of the line connecting two samples indicates the similarity between the samples (short line: highly similar pattern). Dendrogram generated by Norwegian Microarray Consortium from quantile normalized data.

The result from this method is shown as a histogram (Figure 8C). The white bars show a test statistic for a paired *t*-test of HPC vs SVZ (individual cultures from each human have been paired). The blue line shows what one would expect if there were no real differences (just random, due to technical noise etc), based on certain assumptions (see [30]). The pink bars are what the method estimates as real differences calculated as the difference between the white bars and the blue line). The method estimates that around 6% of the genes are different between SVZ and HPC. Although the method states that there are real differences, such estimates can be highly uncertain (cf e.g [30]).

One could further investigate these differences by some Gene Ontology (GO) analysis and see if the most significant GO categories from this analysis make sense for the cell types in question. This, of course, requires that one knows which GO categories are expected to be relevant here.

We applied GO analysis using the R/Bioconductor (www.bioconductor.org) package *GOstats* [41]. The GO Ontological analysis [41] computes a p-value which is the most interesting (as it sums up all the other statistics). It is derived from the following measurements which are listed in: **Table S3**. **GO Tables.xlsx**. “ExpCount” is the expected number of significant genes in the GO category if extra differentially expressed genes are not seen. “Count” is the number of significantly differentially expressed genes in the category. “Size” is the size of the GO category, i.e. the total number of genes in the category. “Odds ratio” is the proportion of significant genes in the GO category divided by the proportion of significant genes overall.

Therefore, to examine whether the differences between the two groups are real, and therefore suggestive of some lineage commitment, we applied a Gene Ontology analysis to see if there were categories of genes upregulated significantly in one group compared to the other, ie. In HPC compared to SVZ. The significant Gene Ontology categories (p<0.05) in each of the three ontologies Biological Process (more than 100 genes), Cellular Component (42 genes), and Molecular Function (73 genes) are listed in: **Table S3. GO Tables.xlsx**.

Because the attempt (**Table S2**) to relate different array platforms and arrays done on different days provoked the suggestion that there were too many sources of error (such as different probe targets, too few genes in common and different technical approaches) to give more than a guide to the relatedness of different adult derived stem cells, we decided to repeat our own arrays such that one platform carried triplicate cultures from three humans for four cell sources (HPC, SVZ, GM and WM) all grown at the same time in identical conditions to the same number of cells and processed together. This was intended to test the hypothesis that these cell populations were identical (Figure 8). The dendrogram method (Figure 8D) cannot clarify whether human individual or tissue region are more significant. Using the fold change greater than three method, cells from grey matter appear to be different to the other three sources (Figure 8A). The *globaltest* analysis (Figure 8B) supports the hypothesis that the differently-derived cell populations are not significantly different (P = 0.77–1.00, n = 3 for each cell source).

### Proteomic Differences

A proteomic comparison was made between SVZ and HPC cultures (mean of three humans for each) for differentially expressed proteins using ‘Silac’ technology that discriminates proteins synthesized in culture incorporating ‘heavy’ amino acids. Data were obtained for 840 proteins. Twelve of these were shown to be upregulated >1.4 fold in SVZ and 11 were shown to be upregulated >1.4 fold in HPC (p<<0.05, n = 3). See Table 2 (and Table S4. HPC(H) to SVZ(L)_Silac.xlsx) for the proteins involved. Column 4 (Table 2) indicates whether this upregulation is reflected in the RNA microarray data. About half were not concordant at message RNA level (Table 2, shaded ratios). One protein (COL1A1, up-regulated in SVZ) was also present within the cohort of most up-regulated genes in SVZ mRNA in our repeated final microarray.

**Table 2:** A proteomic comparison was made between SVZ and HPC cultures.

Protein	Gene Symbol	Protein	mRNA
	Up in SVZ	HPC/SVZ	HPC/SVZ
Ras-related protein R-Ras2	RRAS2	0.40387	0.76
Collagen alpha-1(I) chain	COL1A1	0.40303	0.94
Filamin-B	FLNB	0.53199	**1.01**
Microtubule-associated protein 1B	MAP1B	0.53036	**1.09**
Neurofilament medium polypeptide	NEFM	0.25961	0.93
Collagen alpha-1(XVIII) chain	COL18A1	0.27087	**1.04**
Filamin-C	FLNC	0.55772	0.90
LIM domain only protein 7	LMO7	0.57999	1.00
Heat shock-related 70 kDa protein 2	HSPA2	0.32062	**1.06**
Collagen alpha-2(I) chain	COL1A2	0.60116	1.00
Glycogen phosphorylase, brain form	PYGB	0.63077	0.89
Eukaryotic translation initiation factor 5B	EIF5B	0.69052	**1.03**
	**Up in HCP**		
Mas-related G-protein coupled receptor member F	MRGPRF	2.0517	**0.83**
Phosphate carrier protein, mitochondrial	MCART1	1.7571	1.04
NAD(P)H dehydrogenase [quinone] 1	NQO1	1.7365	1.13
Histone H1.2	HIST1H1C	1.7194	**0.96**
Fatty acid desaturase 2	FADS2	1.7104	**0.90**
Glucose-6-phosphate 1-dehydrogenase	G6PD	1.6318	1.10
Histone H1.5	HIST1H1B	1.4966	**0.99**
Argininosuccinate synthase	ASS1	1.4611	1.26
Collagen alpha-2(VI) chain	COL6A2	1.4224	1.24
Splicing factor, arginine/serine-rich 7	SFRS7	1.3969	**0.96**

A proteomic comparison was made between SVZ and HPC cultures for differentially expressed proteins using ‘Silac’ technology that discriminates proteins synthesized in culture incorporating ‘heavy’ amino acids. Data were obtained for 840 proteins. Twelve of these were shown to be upregulated >1.4 fold in SVZ and 11 were shown to be upregulated >1.4 fold in HPC (p<<0.05, n = 3). Column 4 indicates whether this upregulation is reflected in the RNA microarray data (bolded ratios are those that are not).

### Adult Human Brain Stem Cells are Multipotent

**Figure 9:**
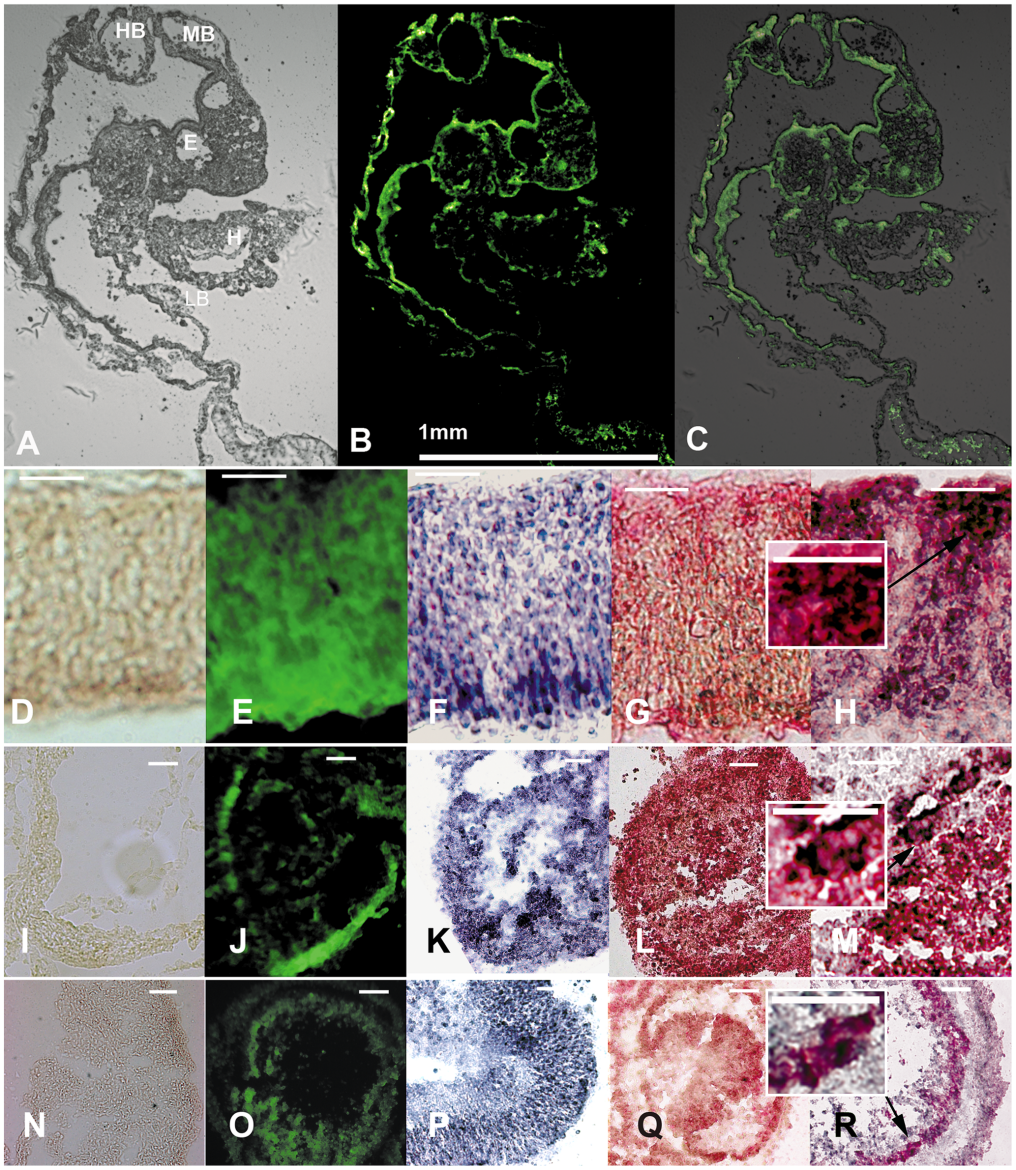
Brain stem cells appear to be multipotent in chick embryo chimeras. Cells were first labeled genetically with a lentivirus expressing GFP under a CMV promoter. Cells were transplanted into the primitive streak of 20 h chick embryos. Top: Descendant cells in embryos harvested at two days later visible under blue excitation as green cells in a variety of tissue locations (C is an overlay of the image in A and the image in B) (HB: hind brain; MB: mid brain; E: eye; H: heart, LB: limb bud). Antibodies against phenotypic markers of the relevant tissues were used to confirm appropriate differentiation of heart muscle (CTNI), striated muscle (sα-actin), and neuronal cells (NF200) (pink stain; G, L, Q). Regions that had shown GFP-positive green human-descended cells (E, J, O) were then also processed for anti GFP-immunochemistry (black stain, F, K, P). As well some sections from regions processed for phenotypic markers (pink) were subsequently co-stained for GFP antigen (black, H, M, R). Insets of high magnification of 100 micron squares depict close proximity of black and pink staining. The black anti-GFP is perinuclear in pattern thus making it highly likely that the two markers are expressed together in individual cells. Panels D, I and N show no primary antibody controls for immunochemistry. Magnification: bars in B, 1 mm; D to R, 100 µm.

Next we tested for multipotence of these cells (three humans, three regions: HPC, SVZ, GM) by transplanting them (in ovo) into the primitive streaks of 20 hour chick gastrulae. Cells were labeled first with a lentivirus expressing GFP under a CMV promoter, or CFDA. Lentiviruses integrate at random and therefore theoretically continue to express GFP in most cell descendants with minimal silencing. Descendant cells in 15 embryos harvested at 2–4 days were visible under blue excitation as green cells in a variety of tissue locations (Figure 9B, C, E, J, O). Regions that had shown GFP-positive green human-descended cells were then also processed for anti GFP-immunochemistry confirming presence of this protein (Figure 9F, K, P). Phenotypic markers of the relevant tissues (specific antibodies against markers of striated muscle, heart muscle and neuronal cells) were used to confirm appropriate differentiation of striated muscle (wing bud), heart muscle and brain vesicle (pink stain; Figure 9C, L, Q). Sections stained for phenotypic markers were subsequently stained with anti-GFP antibody to confirm that some human-derived cells (black stain) co-expressed phenotypic markers (pink stain) of the tissue concerned (Figure 9H, M, R). Insets of high magnification of 100 micron squares depict close proximity of black and pink staining. The black anti-GFP is perinuclear in pattern thus making it highly likely that the two markers are expressed together in individual cells.

That is to say that human adult brain-derived progenitors cultured under these conditions (even at passage 18) developed into striated muscle, heart muscle and neurons following transplantation. This indicates that cells maintained multipotency for up to 18 passages when cultured under these conditions.

### Karyotype of the Cultures Tested

Sample cell cultures were analysed for occurrance of abnormal chromosome content. If one disregards loss of the Y chromosome (a frequent aberration-see Discussion) then results of these analyses are as displayed in Table 3. It can be seen that some cells with abnormal karyotypes occurred in several cultures. In only three passages, one early and two late, were structural aberrations detected. Details of karyotypes observed are described in **Table S5**.

**Table 3:** Karyotype of human-derived brain stem cell cultures.

Patient	Sex	Age	Source	P	Karyotype Early	P	Karyotype	Late
H0938	F	40	SVZ	4	25/25 Normal	10	5/16	Normal
H0867	F	22	SVZ+HPC	8	20/20 Normal	13	23/23	Normal
H0949	M	41	HPC	4	7/9 Normal	11	25/25	Normal
H0980	F	53	HPC	3	22/22 Normal	915	10/252/19	NormalNormal
H1004	M	38	HPC	4	21/25 Normal	1115	20/2522/22	NormalNormal
H1004	M	38	SVZ	3	25/25 Normal	101415	24/2411/1118/18	NormalNormalNormal
H0991	F		WM	3	15/24 Normal			
H0991	F		GM	3	21/24 Normal			
H0995	F		HPC	2	24/24 Normal			

Karyotype of human-derived brain stem cell cultures. Ratios are for number of normal cells (not taking into account loss of Y chromosome) out of total number of cells examined.

### Cloning Brain Stem Cells Some Cloned Cells are Multipotent

In order to test whether one single cell could expand into large numbers and as well possess multipotence, we cloned a culture of cells from grey matter (H0991, passage 1) by fluorescence activated cell sorting (FACs). Single cells were distributed to the wells of 96 well plates in culture medium. After 4 weeks 415 out of a total of 960 wells contained more than one cell (43.3%), 42 were semi-confluent (4.3%) and 42 were confluent. The 42 confluent were replated in 42 much larger wells and of these 29 cultures became confluent again in seven weeks. From these results one can deduce that about 3% of cells (in culture) are long term progenitor cells. One culture was counted and shown to have undergone 17 doublings in 77 days generating 160000 cells. This equates to a population doubling time of 4.5 days. An early sample of cells from this latter culture were labeled *in vitro* with CFDA and then transplanted into three chick gastrulae. After four days embryos were processed and examined for fluorescence. Labelled cells were evident in numerous tissue locations (not shown). Regions of green labelled cells were processed and counterstained positive for striated muscle α-actin (in limb bud), cardiac troponin I (in heart muscle) and neurofilament 200 (in brain vesicle wall).

### Normal Brain Stem Cells do not make Tumours

Cells from these human brain-derived cultures were transplanted into SCID mice in order to ascertain any tendency towards tumor generation. Six animals were transplanted in the brain. Three were euthanased after three months and three more after six months. Sectioning and examination of their brains revealed no evidence of tumor formation.

### Attempts at Differentiation Towards Dopaminergic Phenotype

Many attempts have been made to differentiate a variety of stem and progenitor cells to express a dopaminergic phenotype as measured by percentage of cells positive for tyrosine hydroxylase (TH). Conditions used to successfully differentiate olfactory neuronal stem cells [40] were attempted as well as variations on that theme (see **Materials and Methods**).

**Figure 10:**
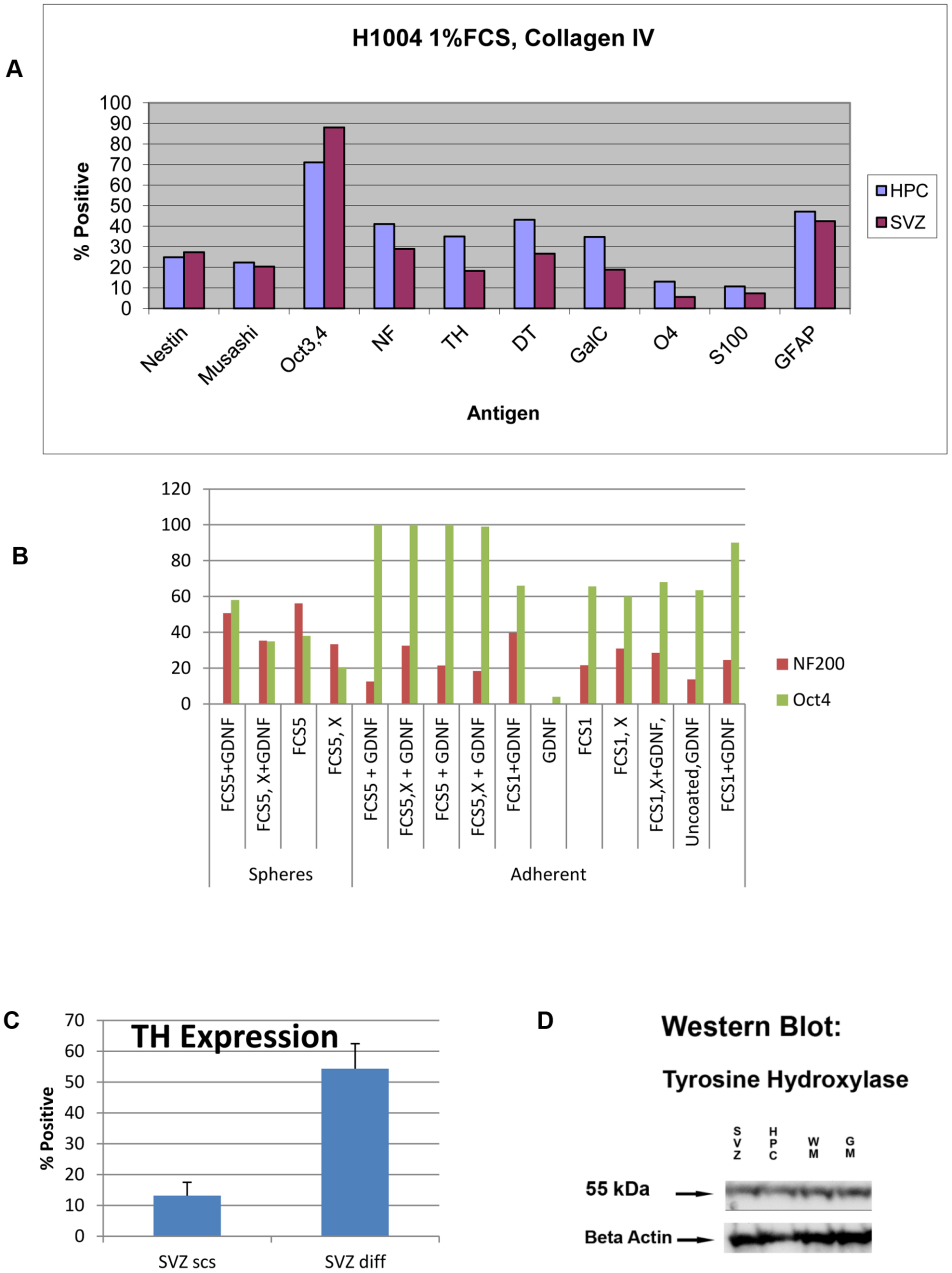
Attempts at differentiation towards a dopaminergic phenotype. **A:** Conditions resulting in high production of TH were 1% FBS in ‘ITS’ on a substrate of Collagen IV where almost 40% of cells were neurons (NF200+) expressing both TH and DT. However these were not really mature cells as the cultures still expressed a high level of the stem cell marker Oct 4 (although Nestin and Musashi were reduced to about 20%). **B:** Highest percentage of neurons (expressing NF) as well as low Oct4 as measures of cellular maturity were attained with 5% FBS, on PLO/Laminin substrate grown directly from neurospheres. FBS5, 5% FBS; FBS1: 1%FBS; X: no FBS; GDNF: 25 ng/mL GDNF. **C**: Highest percentage of TH expressing cells was obtained with 3.5% FBS, on PLO/Laminin substrate grown from adherent stem cells. Three cultures were assessed for each condition. SVZ scs: stem cells derived from SVZ; SVZ diff: cells subjected to putative differentiation conditions. (Error bars: +SEM; n = 3). **D:** Western blot confirms protein expression of TH in ‘differentiated’ cells derived from four region sources of stem cell cultures from one human. 40 µg total protein was loaded in each lane.

Conditions resulting in high production of TH were 1% FBS in ‘ITS’ on a substrate of Collagen IV (Figure 10A) where almost 40% of cells were neurons (NF+) expressing both TH and dopamine transporter (DT). However the same figure (A) indicates that these were not really mature cells as the cultures still expressed a high level of the stem cell marker OCT4 (although Nestin and Musashi were reduced to about 20%).

If one peruses the range of conditions attempted seeking only a high percentage expression of NF as well as low OCT4 as measures of cellular maturity then 5% FBS, PLO/Laminin grown directly from neurospheres resulted in the greatest differentiation (Figure 10B).

Highest percentage of TH expressing cells was obtained with 3.5% FBS, on PLO/Laminin substrate grown from adherent stem cells. Three cultures were assessed for each condition. SVZ stem cells are compared to SVZ cells subjected to putative differentiation conditions (Figure 10C, Bars: SEM; n = 3). When these conditions were applied to neural stem cell cultures derived from Hippocampus, Subventricular Zone, Grey Matter and White Matter of the same individual ‘differentiated’ cultures expressed TH. Western blots of all these cultures confirmed significant expression of this protein (Figure 10D).

## Discussion

It is a widely held hope and an aim of our lab to use cell transplant for treating damaged brain. Injury or degeneration brought about by, for instance Parkinson’s disease or Alzheimer’s disease is a relevant situation [42]. One may envisage that autologous transplant, using the patient’s own cells would provide the best option for a number of reasons. Therefore there should be a benefit in observing and assessing the normal behavior of the endogenous stem cell from the organ affected. For some years it was recognised that cells from the hippocampus and subventricular zone adjacent to the brain ventricles had the ability to give rise to ‘neurospheres’ when cultured in serum free conditions where mitogens such as EGF or bFGF were applied [43], [44]. The capacity of cultures to generate neurospheres (mostly rodent experiments) led to the inference that production of neurospheres was a measure of the existence of a ‘stem’ cell resident in certain specific organic environmental niches [43], [45]. There was controversy over what the properties of this progenitor cell might be. Generally a concept of a ‘primitive’ but lineage committed tissue-specific stem cell arose (many personal communications). Some use the word ‘lineage’ to mean cell of origin; others to mean differentiation restriction; others mean positional history. The context here is the widespread paradigm of ‘differentiation restriction’ which has been well refuted nowadays. Multipotence of adult derived stem cells has been well established [39], [46], [47], [48], [49], [50], [51]. This notion of restricted potential is unjustified in vertebrates where cell development is largely regulative. It was derived from studies of early embryogenesis in ascidians [52]. There has been much speculation about how lineage restriction might take place and indeed all known mechanisms of gene regulation of expression have been invoked though how these mechanisms result in stable specific cell types that still enable the ramifications of tissue differentiation is not clear [53], [54].

Nonetheless optimism for cellular repair by transplantation has appeared boundless with many models tested. Transplant of cells derived from another species [55], [56], another organism of the same species [57], seemingly inappropriate tissue origin [58], biochemically damaged/modified [59] and even genetically transfected cells [60] have all been attempted despite the developmental organisational hurdles believed by so many others [61]. The concept of ‘pluripotency’ has been heralded despite the consequences of potential teratomas [62]. ‘Pluripotent’ means capability to make every cell type of an individual organism except extraembryonic tissues [53]. Experiments have been attempted where pluripotent embryonic stem cells have been transplanted for tissue repair. Tumors often result [63]. As well immune rejection of non-autologous cells is a serious issue [64].

The idea that ‘induced pluripotent stem cells’ (iPS) [65] will solve technical impediments has been embraced enthusiastically by many [66]. Introduction of a plasmid (or four) expressing transcription factors (Oct 4, Sox 2, Klf-4 and c-Myc, originally) ubiquitously, appears to induce a state of ‘pluripotency’ from normally occurring somatic cell types. The purity of mechanistic intervention entailed in introducing a plasmid, inducing a gene’s expression, observing a change in cellular behavior [67] is very useful for defining individual regulatory mechanisms and as well strikes many as the road to explaining how cells and development work. The perceived hurdles of ‘lineage restriction’ can be overcome [68].

It should be a useful strategy to observe and measure the way cells behave and then infer some rules. Not so long ago one could not grow mammalian cells [69], [70], certainly not normal human cells [71], and stem cells in adult tissues were considered unlikely [72]. Failure to attempt sufficient condition variations was interpreted to imply incapacity. The fact is that human organisms can heal damaged organs and tissues well into old age. Many possible cellular defects can occur but even these do not prevent healing within normal limits. It seems conceivable that a general primitive highly programmable cell will be found all throughout the adult human body.

Under the environmental conditions published here we have found that stem cells grow from any biopsy we get. We have successfully cultured the cell populations described in this paper even from minute fragments of tissue yielding an initial cell count of 50000 (mostly differentiated and dying cells). We have been able to expand enormous numbers of cells. A high percentage of these cells expresses OCT 4 and SOX 2, supposed markers of pluripotency. There was no need to ‘induce’ with these transcription factors. Their expression occurred naturally. The rate of occurrence vastly outnumbers ‘induction’ [65], [73]. Interestingly the natural occurrence within cultures targeted for ‘induction’ was not ruled out [66]. The brain stem cell we have described has the ability to generate vast numbers of cells, differentiate into neurons, astrocytes and oligodendrocytes. As well we have assayed its potential to cross into other lineages by transplanting into the early chick embryo. Cells that normally make neural cell types were also able to make cardiac muscle cells and skeletal muscle cells. This potentiality could be discerned in clones (single cells) and we were able to infer percentage presence of primitive cells per different regions assayed. Assessment of phenotype of the various populations was undertaken using immunochemistry, microarray, proteomics and western blot. Thus information was acquired at transcript and protein level. The cell populations, though appearing very similar at gross level, when investigated at a more in depth level (microarray and proteomics) appear to have diverged somewhat in lineage according to region originally sampled but this is by no means clear and there are many potential technical caveats in making this conclusion.

With regard to microarray data we used four methods to appraise whether differences between the different brain region-derived stem cells were real. We applied *J-Express* fold change tool to tabulate the number of genes differing by more than three-fold between cell populations. We applied *locfdr* [30] to test whether the hippocampus (HPC) and subventricular (SVZ) expression values are statistically different from each other. We applied GO analysis [41] to these results. Later the method *globaltest* [31] was applied to reappraise statistical differences over four brain stem cell sources.

We performed GO analysis [41] to see if genes up-regulated in HPC-derived cells compared to SVZ-derived cells gave insight into or evidence of ‘lineage’. GO categories where genes were upregulated significantly in HPC compared to SVZ (p<0.05) in each of the three ontologies Biological Process (more than 100 genes), Cellular Component (42 genes), and Molecular Function (73 genes) appear to indicate that the cells really are taking on some functional differentiation at this stage of cellular state. This data is presented for interest (**Table S3. GO Tables.xlsx**).

An assessment of the karyotypes in early and later cultures revealed some genomic instability. There are many studies showing karyotypic abnormalities in neoplastic tissue [74] and the finding of such genomic aberrations in tumors are thought of as proof of neoplastic disease. Human *in vitro* cultures of neural stem cells [75] showed karyotypic instability whilst olfactory stem cells did not [29]. Reports from *in vitro* fertilisation clinics attest greater than 50% chromosomal abnormality in *in vitro*-derived embryos [76]. Evidence thus suggests that instability of chromosomal organisation must be viewed as a relevant possible danger leading to *in vitro* clonal selection of aberrant cell populations. Any potential use for tissue repair will therefore require stringent assessment and quality control of *in vitro*-derived cells.

Most of our stem cell cultures were karyotypically quite stable, but some genomic instability was seen. The aberration most often encountered was loss of the Y chromosome, a finding seen not only in neoplastic tissue, but also in elderly men and in some presumably non-neoplastic tissues [77]. The latter goes also for trisomy 7 seen in one sample. On the other hand, structural, unbalanced aberrations found in three passages are clearly different and must be thought of as having a neoplastic potential.

There was evidence of some clonal instability with karyotypically normal early passage and abnormal late passage, as well as abnormal early passage and normal late passage. Independent clones were also observed in several cell line passages and in one cell line stepwise accumulation of chromosomal aberrations, clonal evolution, could be suspected. Although chromosomal aberrations were found in some early cell line passages, it seems most likely that the abnormalities observed are *in vitro* generated. Therefore, it is important to keep track of the genomic status of the cell lines at different passages. Such genomic changes can potentially lead to different and/or unexpected phenotypic changes.

We have experimented at length to try to induce differentiation in these cells with a particular emphasis on obtaining a dopaminergic phenotype. Some of our results have been included in Figure 10. Under certain conditions 50% of cells could be induced to express dopaminergic and neural markers whilst showing a diminution in the percentage of cells expressing stem cell markers.

A glimpse of the manipulations we made suggests the difficulties of bringing about bona fide expression of a natural cell type *in vitro*. The precise conditions enabling tissue development are yet to be well understood [53]. But we are aware of a multitude of individual mechanisms and a synthesized understanding of development will eventually be achieved. It may be elusive requiring new concepts in understanding [54], not necessarily a clockwork mechanistic interpretation [78].

To sum up then we have shown that similar to many other body sources, abundant primitive progenitor cells (stem cells) can be derived from the human brain. These are easy to propagate, have markers of pluripotency as well as some apparent lineage leanings and are capable of being induced to provide descendants in other lineage compartments. Much work is needed yet to understand the management of adult stem cells and whether a source of neural stem cells such as the filum terminale or the external nares could provide a cell source for brain repair. But on the basis of our data presented here it seems likely that stem cells gleaned from a white matter biopsy for instance might well provide a source to repair another structure such as hippocampus.

## Supporting Information

Figure S1 **Phenotype of brain stem cell cultures.** **Stratification of cultures.** Cultures could be arbitrarily divided into zones based on the appearance of the cells in these zones. Immunophenotype confirmed that the differences in shape also reflected differences in phenotype and suggest that this stratification in some way reflects the dynamics involved in tissue organisation. Each panel across has an **Inner zone**, an **Intermediate zone,** and an **Outer zone** with representative cell types shown. The first photo in each panel has the symbol of the protein antigen depicted in each of the three zones. **Putative markers of cell types: Positive control**: Prol4OHase (Proline-4-hydroxylase ubiquitous subunit) **Dividing cells:** Ki67; **Stem cells**, Oct 4, Sox2, IntB1 (Integrin β1) EGFr, Mus (Musashi), Nestin. **Astrocytes,** GFAP, S100. **Neurons**: BTub3 (βTubulin3), Map2, NF. **Oligodendrocytes:** O4, GalC. **Dopaminergic cells**: TH, DT. First panel depicts **Secondary antibody only** negative controls. Bars: 100 µm.
(PDF)

Figure S2 **Double staining using immunofluorescent secondary antibodies.** Red is Alexaflor 594, Green is Alexaflor 488. Where antigens are closely associated red and green together appear yellow/orange. Where antigens are nuclear they merge to give pink (for red). Oct4 co-localizes with GFAP often, sometimes with NF and TUBB3 (βTubulin 3). Nestin co-localizes often with GFAP, sometimes with TUBB3 and sometimes O4. O4 sometimes co-localizes with TUBB3. TH can co-localize with DT. TH can co-localize with TUBB3. NF can co-localize with DT. Bars: 100 µm.
(PDF)

Figure S3 **Neurospheres in suspension culture.** Neural stem cells were grown adherently, labelled with lentivirus to express GFP and then induced to grow as neurospheres (See Materials and Methods) which then grew in suspension culture. Bar: 100 µM.
(PDF)

Table S1 **Constituents by marker in spheres compared to adherent cultures.**
(DOCX)

Table S2 **Two-way table allowing inference of relatedness (number of genes differing more than three-fold in expression, less = closer) between various human adult ‘stem’ cell types).** Arrays published are from different platforms and times and have been ‘normalised’ by a statistician (See Materials and Methods). HPC: hippocampus; SVZ: Subventricular zone; GM: grey matter; WM: white matter; MSC: mesenchymal stem cell; NSP: neurospheres (cultured from SVZ); OSC: olfactory stem cell; TSCad: Glioblastoma stem cells (adherent culture); TSPs: Glioblastoma stem cells (neurosphere culture); SVZsp: Subventricular zone (neurospheres after adherent culture). Unless otherwise stated cells used were cultured adherently. Total number of genes in this comparison: 7264.
(DOCX)

Table S3 **GO Tables.xlsx.** Gene Ontology inference from microarray data mining of Subventricular zone- and Hippocampus-derived cultures.
(XLSX)

Table S4 **HPC(H) to SVZ(L)_Silac.xlsx).** Details of raw ‘Silac’ data.
(XLSX)

Table S5 **Actual karyotypes.** Sample cell cultures were cultured, harvested, G-banded using Wright stain, and a karyotype established [35], [36]. Of the three cell lines where only early passages were examined, two had abnormal karyotypes (one numerical aberration each) and one was normal. Both early and late passages were analyzed for six stem cell cultures; in one of the cultures both passages were normal, in one culture the early passage was abnormal and the late normal, in two cultures all passages were abnormal, and in two cultures the early passage was normal and the late passage abnormal. Most aberrations were numerical and loss of the Y chromosome was the most frequent aberration. In only three passages, one early and two late, structural aberrations were detected.
(DOCX)
