# Supplementary material for: Expansion of Multipotent Stem Cells from the Adult Human Brain
Source: PLoS One. 2013 Aug 14;8(8):e71334. doi: 10.1371/journal.pone.0071334 (PMC3743777; doi:10.1371/journal.pone.0071334)
Supplement: Figure S1 — Phenotype of brain stem cell cultures. Stratification of cultures. Cultures could be arbitrarily divided into zones based on the appearance of the cells in these zones. Immunophenotype confirmed that the differences in shape also reflected differences in phenotype and suggest that this stratification in some way reflects the dynamics involved in tissue organisation. Each panel across has an Inner zone, an Intermediate zone, and an Outer zone with representative cell types shown. The first photo in each panel has the symbol of the protein antigen depicted in each of the three zones. Putative markers of cell types: Positive control: Prol4OHase (Proline-4-hydroxylase ubiquitous subunit) Dividing cells: Ki67; Stem cells, Oct 4, Sox2, IntB1 (Integrin β1) EGFr, Mus (Musashi), Nestin. Astrocytes, GFAP, S100. Neurons: BTub3 (βTubulin3), Map2, NF. Oligodendrocytes: O4, GalC. Dopaminergic cells: TH, DT. First panel depicts Secondary antibody only negative controls. Bars: 100 µm. (PDF) [file pone.0071334.s001.pdf]

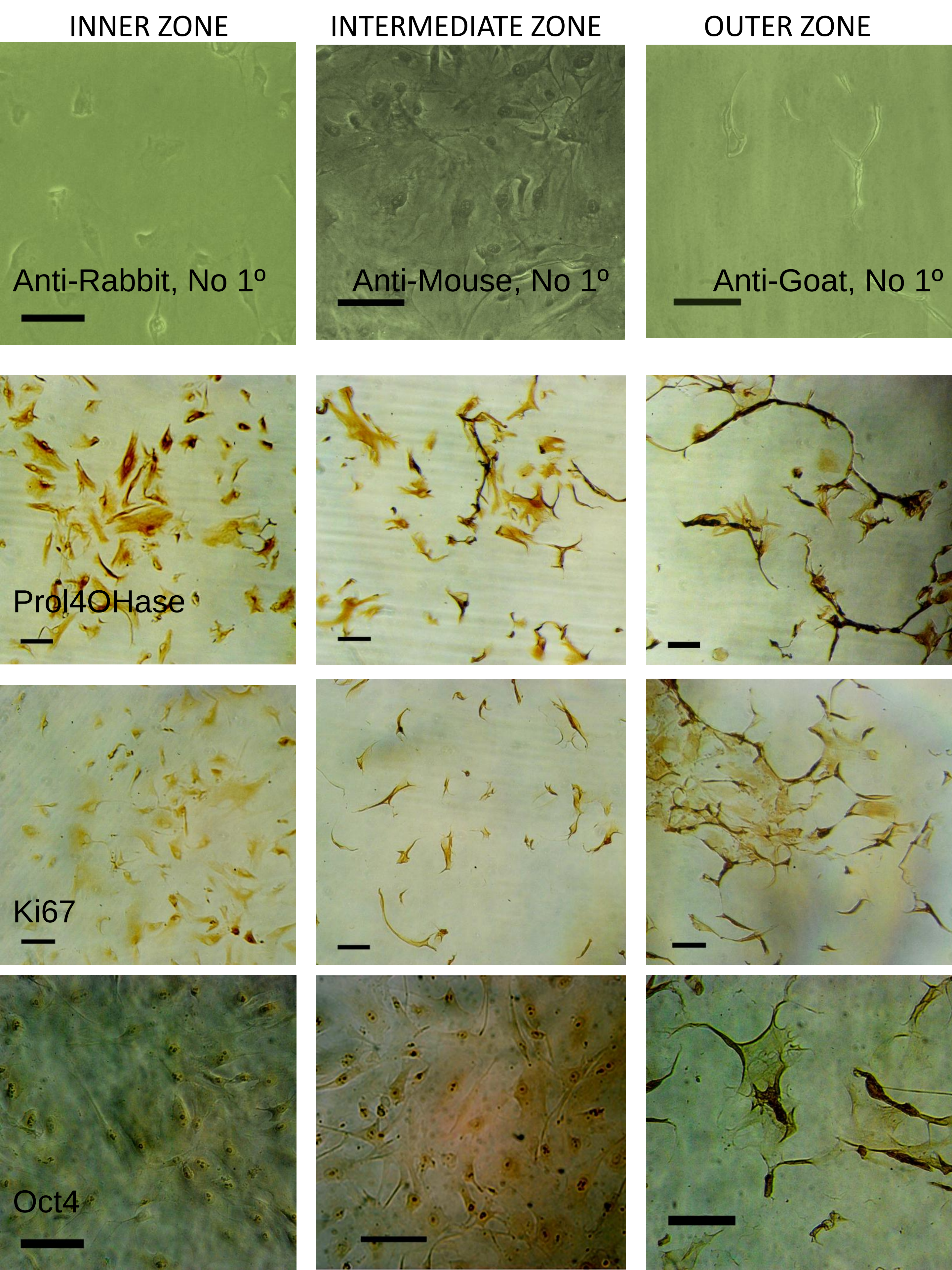
INNER ZONE
INTERMEDIATE ZONE
OUTER ZONE
Anti-Rabbit, No 1º
Anti-Mouse, No 1º
Anti-Goat, No 1º
Prol4OHase
Ki67
Oct4

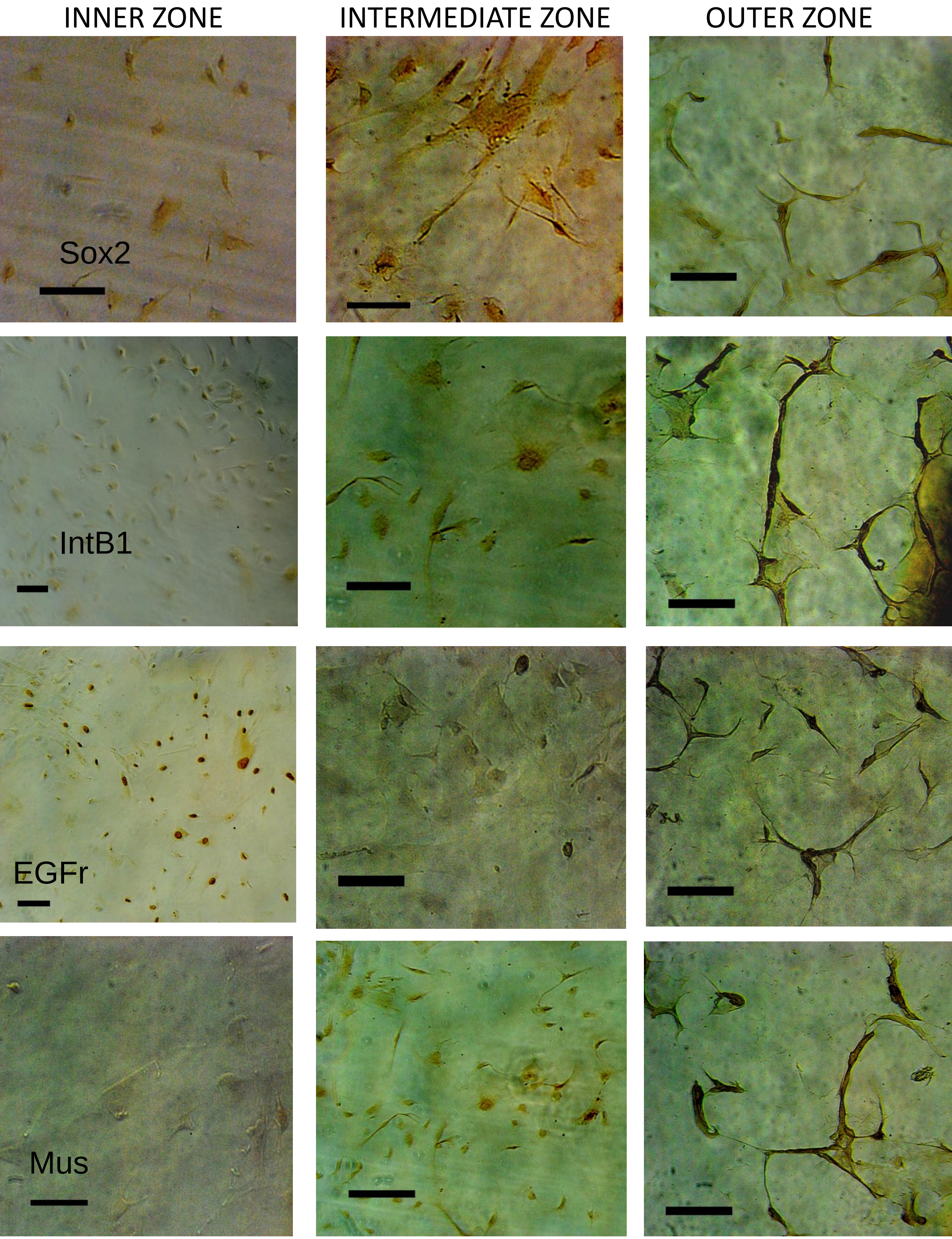
INNER ZONE
INTERMEDIATE ZONE
OUTER ZONE
Sox2
IntB1
EGFr
Mus

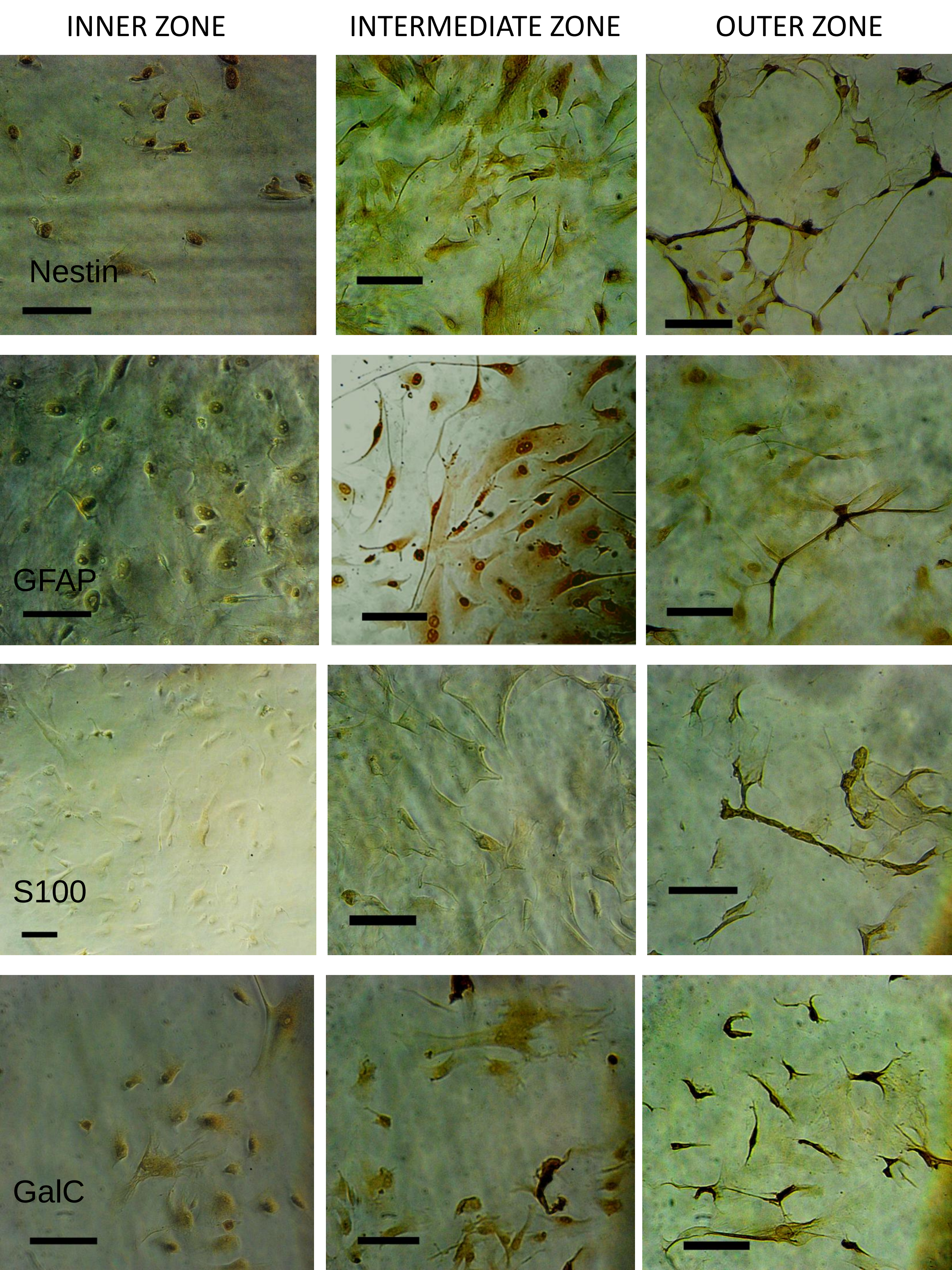
INNER ZONE
INTERMEDIATE ZONE
OUTER ZONE
Nestin
GFAP
S100
GalC

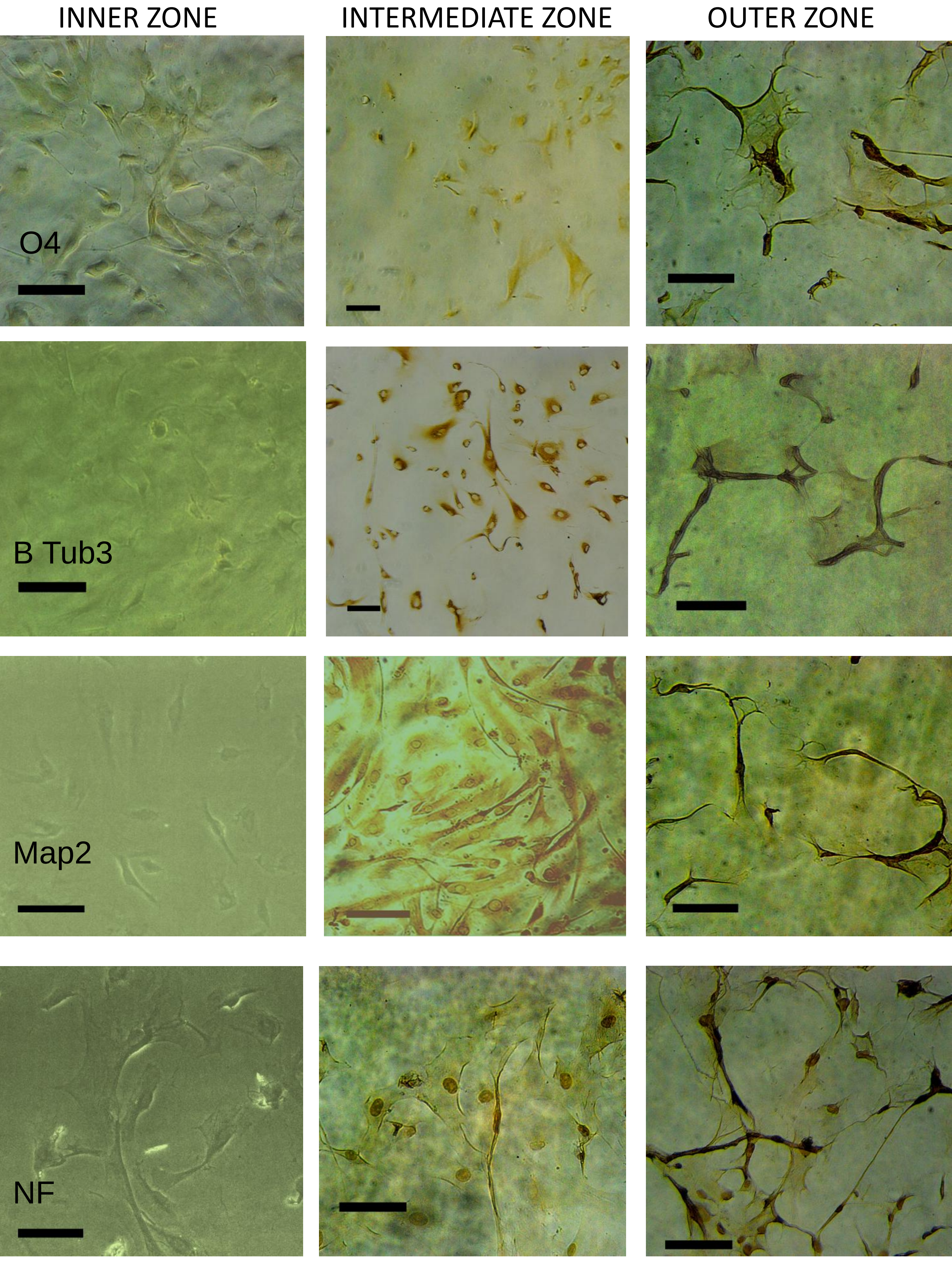

INNER ZONE
INTERMEDIATE ZONE
OUTER ZONE
O4
B Tub3
Map2
NF

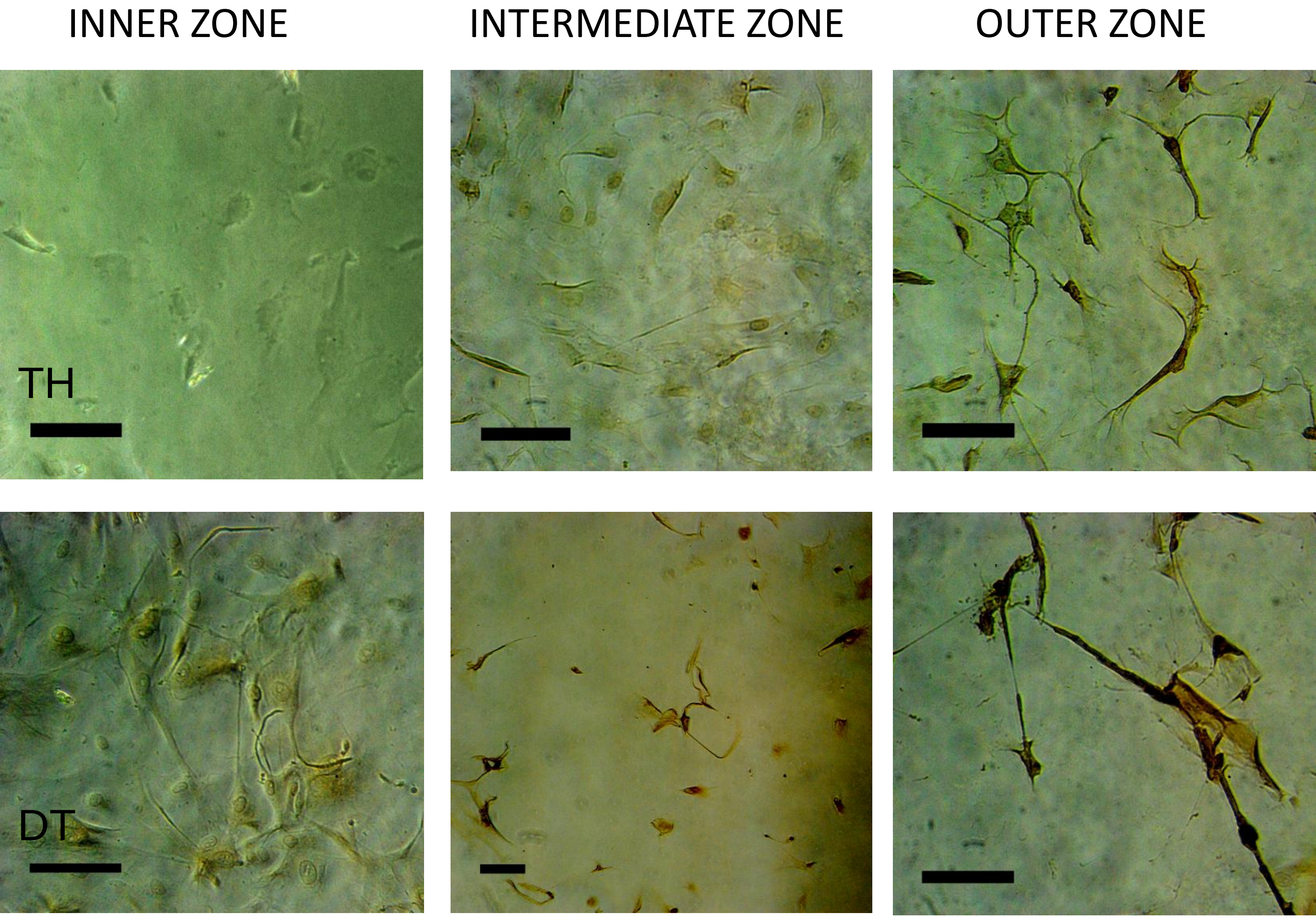
INNER ZONE
INTERMEDIATE ZONE
OUTER ZONE
TH
DT
