# Supplementary figures and images for: Expansion of Multipotent Stem Cells from the Adult Human Brain
Source: PLoS One. 2013 Aug 14;8(8):e71334. doi: 10.1371/journal.pone.0071334 (PMC3743777; doi:10.1371/journal.pone.0071334)

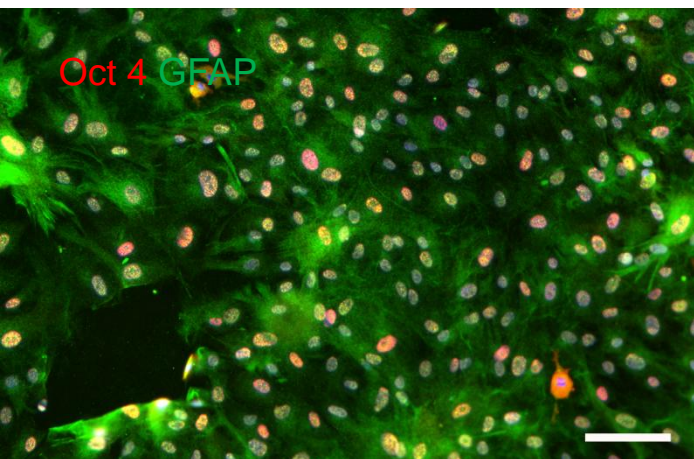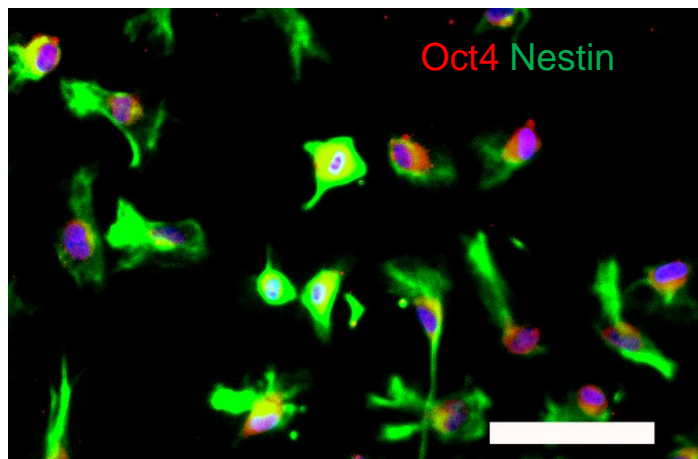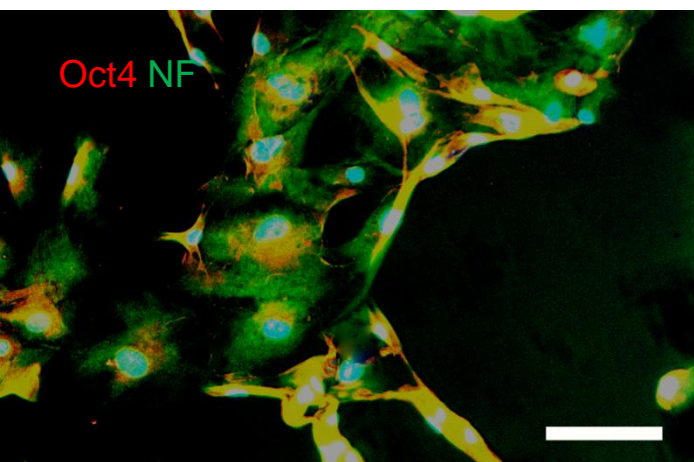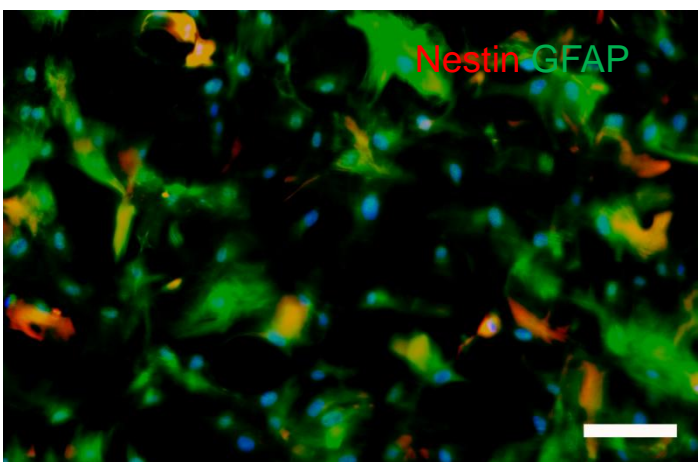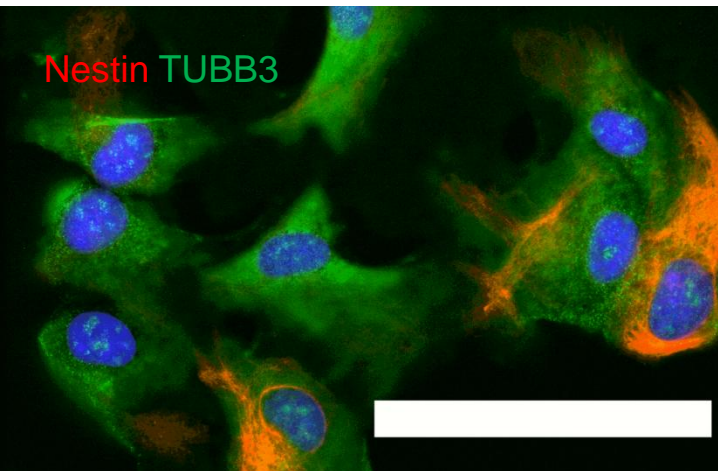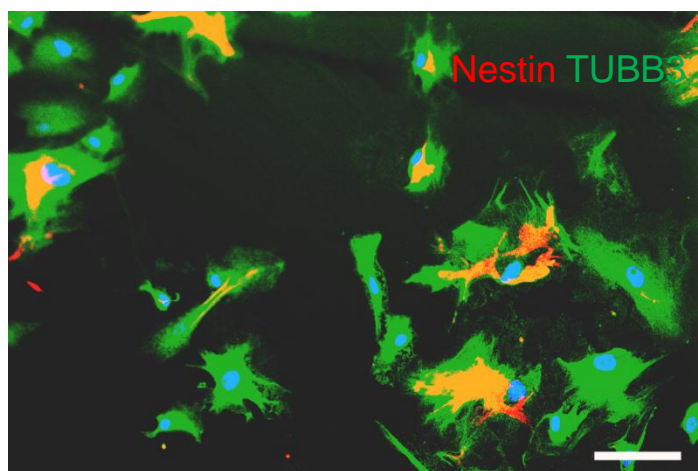

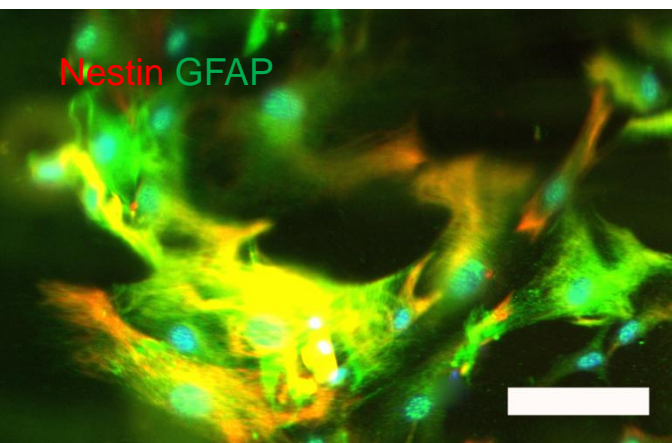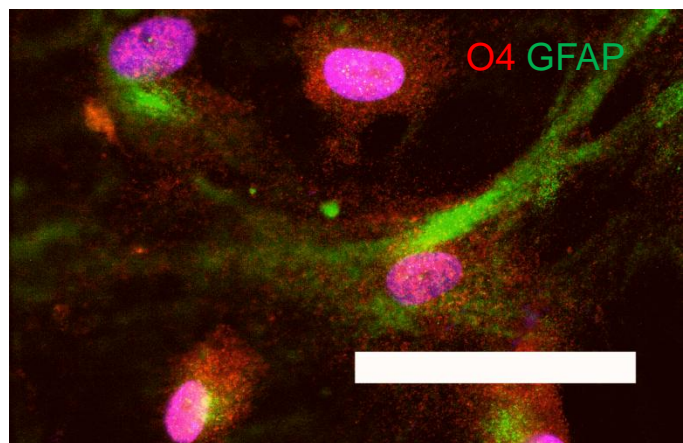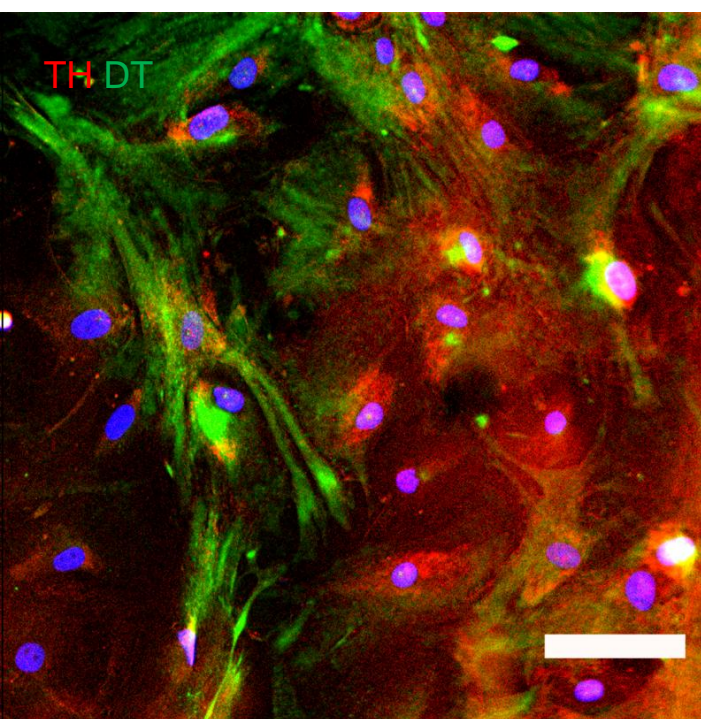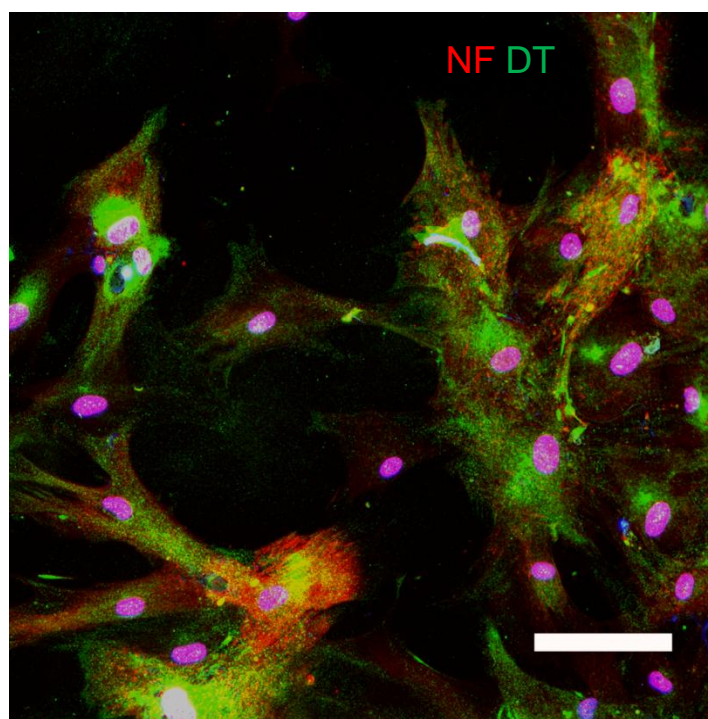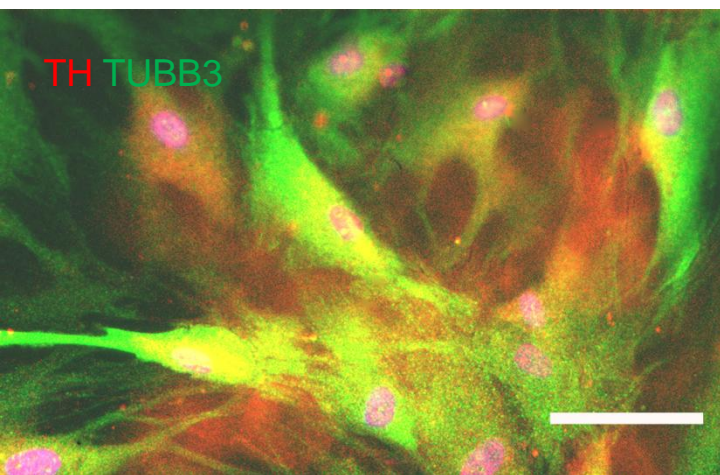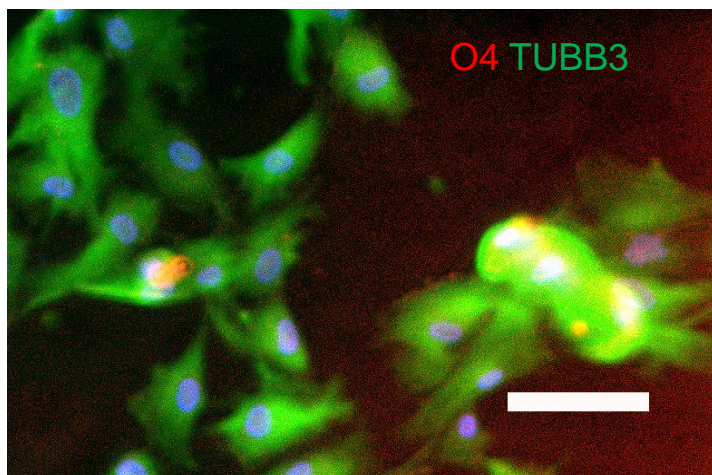

Supplement: Figure S2 — Double staining using immunofluorescent secondary antibodies. Red is Alexaflor 594, Green is Alexaflor 488. Where antigens are closely associated red and green together appear yellow/orange. Where antigens are nuclear they merge to give pink (for red). Oct4 co-localizes with GFAP often, sometimes with NF and TUBB3 (βTubulin 3). Nestin co-localizes often with GFAP, sometimes with TUBB3 and sometimes O4. O4 sometimes co-localizes with TUBB3. TH can co-localize with DT. TH can co-localize with TUBB3. NF can co-localize with DT. Bars: 100 µm. (PDF) [file pone.0071334.s002.pdf]

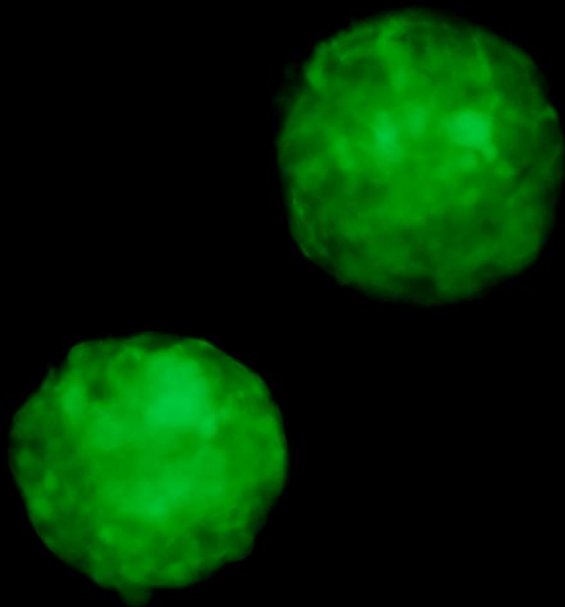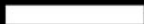

Supplement: Figure S3 — Neurospheres in suspension culture. Neural stem cells were grown adherently, labelled with lentivirus to express GFP and then induced to grow as neurospheres (See Materials and Methods) which then grew in suspension culture. Bar: 100 µM. (PDF) [file pone.0071334.s003.pdf]
