# Supplementary material for: Expansion of Multipotent Stem Cells from the Adult Human Brain
Source: PLoS One. 2013 Aug 14;8(8):e71334. doi: 10.1371/journal.pone.0071334 (PMC3743777; doi:10.1371/journal.pone.0071334)
Supplement: Table S1 — Constituents by marker in spheres compared to adherent cultures. (DOCX) [file pone.0071334.s004.docx]

**Table S1. Neurospheres and adherent cultures.**

|  | **Stem cells** | **Neural stem cells** | **Neurons** | **Astrocytes** | **Oligodendrocytes** |
| --- | --- | --- | --- | --- | --- |
| **Marker** | Sox2 | Nestin | TUBB3 | GFAP | O4 |
|  |  |  |  |  |  |
| **Brain neurospheres (adult Human)(Slices)** Vik-Mo (unpublished) | 35% | 49% | 20% | 77% | 32% |
|  |  |  |  |  |  |
| **Olfactory neurospheres (adult Rat)** **(Dissociated )** Wetzig et al[2011] | Not done | 48% | 17% | 79% | Not done |
|  |  |  |  |  |  |
| **Brain adherent stem cells (adult Human)** This paper | 32 % | 36 % | 24 % | 36 % | 21 % |
