# Supplementary material for: Expansion of Multipotent Stem Cells from the Adult Human Brain
Source: PLoS One. 2013 Aug 14;8(8):e71334. doi: 10.1371/journal.pone.0071334 (PMC3743777; doi:10.1371/journal.pone.0071334)
Supplement: Table S2 — Two-way table allowing inference of relatedness (number of genes differing more than three-fold in expression, less = closer) between various human adult ‘stem’ cell types). Arrays published are from different platforms and times and have been ‘normalised’ by a statistician (See Materials and Methods). HPC: hippocampus; SVZ: Subventricular zone; GM: grey matter; WM: white matter; MSC: mesenchymal stem cell; NSP: neurospheres (cultured from SVZ); OSC: olfactory stem cell; TSCad: Glioblastoma stem cells (adherent culture); TSPs: Glioblastoma stem cells (neurosphere culture); SVZsp: Subventricular zone (neurospheres after adherent culture). Unless otherwise stated cells used were cultured adherently. Total number of genes in this comparison: 7264. (DOCX) [file pone.0071334.s005.docx]

**Table S2. Different ‘stem cell’ cultures.**

|  | **HPC** | **SVZ** | **GM** | **WM** | **MSC** | **NSP** | **OSC** | **TSCad** | **TSPs** | **SVZsp** |
| --- | --- | --- | --- | --- | --- | --- | --- | --- | --- | --- |
| **HPC** | 0 | 5 | 94 | 66 | 2204 | 2766 | 988 | 920 | 1418 | 726 |
| **SVZ** | 5 | 0 | 249 | 44 | 2164 | 2738 | 957 | 906 | 1391 | 169 |
| **GM** | 94 | 249 | 0 | 32 | 1428 | 1927 | 544 | 794 | 894 | 272 |
| **WM** | 66 | 44 | 32 | 0 | 1377 | 1980 | 524 | 769 | 902 | 353 |
| **MSC** | 2204 | 2164 | 1428 | 1377 | 0 | 2474 | 2010 | 2174 | 2463 | 1393 |
| **NSP** | 2766 | 2738 | 1927 | 1980 | 2474 | 0 | 2769 | 2592 | 2549 | 2670 |
| **OSC** | 988 | 957 | 544 | 524 | 2010 | 2769 | 0 | 1084 | 1488 | 1107 |
| **TSCad** | 920 | 906 | 794 | 769 | 2174 | 2592 | 1084 | 0 | 401 | 680 |
| **TSPs** | 1418 | 1391 | 894 | 902 | 2463 | 2549 | 1488 | 401 | 0 | 1126 |
| **SVZsp** | 726 | 169 | 272 | 353 | 1393 | 2670 | 1107 | 680 | 1126 | 0 |
