# Supplementary material for: Expansion of Multipotent Stem Cells from the Adult Human Brain
Source: PLoS One. 2013 Aug 14;8(8):e71334. doi: 10.1371/journal.pone.0071334 (PMC3743777; doi:10.1371/journal.pone.0071334)
Supplement: Table S5 — Actual karyotypes. Sample cell cultures were cultured, harvested, G-banded using Wright stain, and a karyotype established [35], [36]. Of the three cell lines where only early passages were examined, two had abnormal karyotypes (one numerical aberration each) and one was normal. Both early and late passages were analyzed for six stem cell cultures; in one of the cultures both passages were normal, in one culture the early passage was abnormal and the late normal, in two cultures all passages were abnormal, and in two cultures the early passage was normal and the late passage abnormal. Most aberrations were numerical and loss of the Y chromosome was the most frequent aberration. In only three passages, one early and two late, structural aberrations were detected. (DOCX) [file pone.0071334.s008.docx]

**Table S5. Actual karyotypes.**

H0938p4: 46,XX[25]

H0938 p10: 45,XX,-12,add(12)(p12),add(14)(q32)[cp11]/46,XX[5]

H0949 HPC p4: 45,X,-Y[6]/47,XY,+7[2]/46,XY

H0949 HPC p11: 46,XY[25]

H0980 HPC p3: 46,XX[22]

H0980 p9: 45,X-X[11]/46,XX,add(1)(q32)[4]/46,XX[10]

H0980 p15: 35-85,inc[17]/46,XX[2]

H1004 HPC p4: 45,X-Y,[6]/48,XY,+?8,+20[cp4]/46,XY[15]

H1004 HPC p11: 45,X,-Y[13]/46,X,-Y,+20[2]/46~47,XY,+?8[3]/46,XY[7]

H1004 HPC p15: 45,X,-Y[11]/46,XY[11]

H1004 SVZ p3: 45,X,-Y[11]/46,XY[13]

H1004 SVZ p10: 45,X,-Y[22]/46,XY[2]

H1004 SVZ p14: 45,X,-Y[3]/46,XY[8]

H1004SVZ p15: 45,X,-Y[18]

H0867p8: 46,XX[20]

H0867p13: 46,XX[23]

H0991 WM p3: 45,X,-X[9]/46,XX[15]

H0991 GM p3: 47,XX,+8[3]/46,XX[21]

H0995 HPC p2: 46,XX[24]
